# Microbial community composition in the rhizosphere of *Pteris vittata* and its effects on arsenic phytoremediation under a natural arsenic contamination gradient

**DOI:** 10.3389/fmicb.2022.989272

**Published:** 2022-09-06

**Authors:** Pu Jia, Fenglin Li, Shengchang Zhang, Guanxiong Wu, Yutao Wang, Jin-tian Li

**Affiliations:** ^1^Guangdong Provincial Key Laboratory of Biotechnology for Plant Development, Guangzhou Key Laboratory of Subtropical Biodiversity and Biomonitoring, School of Life Sciences, South China Normal University, Guangzhou, China; ^2^School of Life Sciences, Sun Yat-sen University, Guangzhou, China; ^3^Dongli Planting and Farming Industrial Co., Ltd., Lianzhou, China

**Keywords:** arsenic, AsChip, bacteria, pathogenic fungi, phytoremediation, *Pteris vittata*, symbiotic fungi

## Abstract

Arsenic contamination causes numerous health problems for humans and wildlife via bioaccumulation in the food chain. Phytoremediation of arsenic-contaminated soils with the model arsenic hyperaccumulator *Pteris vittata* provides a promising way to reduce the risk, in which the growth and arsenic absorption ability of plants and the biotransformation of soil arsenic may be greatly affected by rhizosphere microorganisms. However, the microbial community composition in the rhizosphere of *P. vittata* and its functional role in arsenic phytoremediation are still poorly understood. To bridge this knowledge gap, we carried out a field investigation and pot experiment to explore the composition and functional implications of microbial communities in the rhizosphere of four *P*. *vittata* populations with a natural arsenic contamination gradient. Arsenic pollution significantly reduced bacterial and fungal diversity in the rhizosphere of *P*. *vittata* (*p* < 0.05) and played an important role in shaping the microbial community structure. The suitability of soil microbes for the growth of *P*. *vittata* gradually decreased following increased soil arsenic levels, as indicated by the increased abundance of pathogenic fungi and parasitic bacteria and the decrease in symbiotic fungi. The analysis of arsenic-related functional gene abundance with AsChip revealed the gradual enrichment of the microbial genes involved in As(III) oxidation, As(V) reduction, and arsenic methylation and demethylation in the rhizosphere of *P*. *vittata* following increased arsenic levels (*p* < 0.05). The regulation of indigenous soil microbes through the field application of fungicide, but not bactericide, significantly reduced the remediation efficiency of *P*. *vittata* grown under an arsenic contamination gradient, indicating the important role of indigenous fungal groups in the remediation of arsenic-contaminated soil. This study has important implications for the functional role and application prospects of soil microorganisms in the phytoremediation of arsenic-polluted soil.

## Introduction

Arsenic is one of the most carcinogenic and toxic substances in the world, posing high risks to the health of humans and wildlife via bioaccumulation in the food chain (Oberoi et al., 2019). The inappropriate disposal of mining waste causes arsenic contamination in the surrounding soil and water of mining areas, creating an urgent need for the remediation of arsenic-contaminated habitats, for which a comprehensive understanding of the mechanism is particularly important (Sun et al., 2019a). Contemporary remediation methods for arsenic-contaminated soil mainly include physical, chemical, and biological approaches; the former two have the disadvantages of high cost and the risk of potential secondary contamination (Yao et al., 2012; Gong et al., 2018). The bioremediation of arsenic-contaminated soil can be performed by microbial remediation and phytoremediation (Jatav and Singh, 2015; Tian et al., 2021). Phytoextraction and phytostabilization are the two main phytoremediation strategies that have proven to be more suitable and eco-friendly for the remediation of large areas and diffuse and surface-contaminated soils (Chaney and Baklanov, 2017). Microorganisms can remediate arsenic-contaminated soils through a variety of reactions, including arsenic adsorption, oxidation and reduction, leaching, transformation, and volatilization (He et al., 2020). The complex feedback between plants and rhizosphere microbes makes microbes the key determinants in facilitating phytoremediation (Li et al., 2018; Jia et al., 2020). Although the effects of phytoremediation and microbial remediation can be disturbed by factors such as regional climate, they have been considered the most cost-effective technology for the current remediation of arsenic-contaminated soils (Gong et al., 2018). Therefore, exploring microbe-assisted phytoremediation mechanisms has great value for further developing and improving methods for the remediation of soil arsenic contamination.

Phytoremediation takes advantage of the characteristics of accumulator plant(s) to naturally absorb toxic metals in soil to achieve the remediation of soil contamination (Behera, 2014). Phytoextraction, as a low-cost, environmentally friendly, and sustainable strategy, has been widely studied for decades (Li et al., 2009, 2017). The perennial fern *Pteris vittata* (Chinese brake fern) was the first known arsenic hyperaccumulator to be highly tolerant and efficient in accumulating arsenic. *Pteris vittata* has been reported to accumulate up to 22,630 mg kg^−1^ dry weight of arsenic in fronds (Ma et al., 2001), arousing extensive research interest in its application in the phytoremediation of arsenic-contaminated soil (Xie et al., 2009; Xiao et al., 2021). Studies have shown that the root exudates of *P*. *vittata* can affect soil pH, thereby altering the bioavailability of arsenic in soil (Yang et al., 2022), which is one of the main factors limiting the arsenic absorption efficiency of *P*. *vittata*. In the process of phytoremediation, the synergistic effects of hyperaccumulators and the rhizosphere soil microenvironment, such as soil metabolism, soil fertility, and microbial community structure, can significantly promote the effect and sustainability of soil remediation (Giloteaux et al., 2013). Studies of plant–microbe interactions have shown that plants can shape their own rhizosphere microbial communities, which are complex plant-associated assemblages with tremendous importance for plant nutrition and health (Edwards et al., 2015). For example, a recent report demonstrated that rhizosphere prokaryotic and fungal communities differentially responded to soil properties and plant performance, in terms of both diversity and community structure and in their interactions with host plants, mainly due to the different characteristics of their potential functional guilds (Bai et al., 2022). Another study of the molecular mechanisms of arsenic accumulation in *P*. *vittata* showed that *P*. *vittata* is a major driver of shifts in the structure and function of the rhizosphere soil microbiome. Thus, exploring the synergistic effect of hyperaccumulators on rhizosphere microorganisms can improve our understanding of the roles of plants and associated microbes in metal mobilization (Yang et al., 2022).

Although the arsenic tolerance and accumulation capability of *P*. *vittata* is an inherent attribute, its remediation efficiency largely depends on the performance of rhizosphere microorganisms (Zhu et al., 2014). Arsenic exists in soils mainly as pentavalent arsenic [As(V)] and trivalent arsenic [As(III)], and microorganisms play an important role in the accumulation and transformation of arsenic. In plant, the toxicity of trivalent arsenic [As(III)] is greater than that of pentavalent arsenic [As(V)], while the toxicity of inorganic arsenic is greater than that of organic arsenic (Zhu et al., 2014). Microorganisms are mainly involved in arsenic metabolism and biological transformation in soil through their functional genes related to arsenic cycling (Abou-Shanab et al., 2022). The arsenic metabolism mediated by microbial functional genes, including arsenic redox (*aoxR*, *arxA*, *aioAB*, *and arsJ*), reduction (*arsC*, *gstB*, *and arrAB*), methylation and demethylation (*arsI* and *arsM*), and efflux (*arsP*, *arsK*, and *arsB*), is a determinant of arsenic bioavailability in soil and an important step of global arsenic geochemical cycling (Zhu et al., 2017; Chen et al., 2020) that has been extensively reviewed (Zhu et al., 2017; Wu et al., 2022a). To explore these key functional genes, Zhao et al. (2018) developed a high-throughput qPCR chip (AsChip) containing 81 primer sets targeting 19 arsenic-related genes, aiming to comprehensively detect those genes linked to the microbial cycling of arsenic. This can be linked with the results of omics analysis to solve problems such as arsenic resistance, biotransformation, and biogeochemistry, as well as quantitative ecological risk assessment. The absorption of arsenic in the soil by *P*. *vittata* involves complex interactions that occur between plants and microbes (Han et al., 2017; Sun et al., 2020), plants and the environment (Ghosh et al., 2015), and microbes and the environment (Ghosh et al., 2015). To fully utilize the role soil microorganisms play in arsenic phytoremediation, it is necessary to fully understand the microbial community composition in the rhizosphere of naturally grown *P*. *vittata* and their functional role in the arsenic accumulation of *P*. *vittata*. Such knowledge, however, is still lacking.

Herein, we carried out a field investigation and pot experiment on four *P*. *vittata* populations grown under a natural arsenic contamination gradient to explore the composition and structure of the rhizosphere bacterial and fungal communities and their impact on the arsenic accumulation ability of *P*. *vittata*. We hypothesized that (i) the soil arsenic level plays an important role in shaping the microbial community structure, and (ii) the effects of arsenic on the microbial community further impact the arsenic accumulation ability of *P*. *vittata*. To our knowledge, this study offers the most comprehensive understanding thus far of the functional role of indigenous soil microorganisms in the phytoremediation of arsenic-polluted soil by *P*. *vittata*.

## Materials and methods

### Experimental design and sample collection

We collected rhizosphere soil samples and *P*. *vittata* samples from four field sites with a natural arsenic-contamination gradient: Chadong arsenic mine, Yunfu City, Guangdong Province (111°59′46.88″E, 22°49′15.21″N; total soil arsenic level: 24488 ± 16,148 mg·kg^−1^, named as heavily); Xiangxiong arsenic mine, Shimen County, Hunan Province (111°02′04.41″E, 29°39′10.13″N; total soil arsenic level: 618 ± 2,738 mg·kg^−1^, named as moderately arsenic-contaminated); Lutang tailings pond, Dachang mining area, Guangxi Province (107°34′25.60″E, 24°49′15.54″N; total soil arsenic level: 1989 ± 1893 mg·kg^−1^, named as weakly arsenic-contaminated); and Heishiding Nature Reserve, Zhaoqing City, Guangdong Province (111°54′13.06″E, 23°28′12.12″N; total soil arsenic level: 110.40 ± 1.40 mg·kg^−1^, named as non-arsenic-contaminated; Supplementary Figure S1). Site Chadong is an exposed arsenic mine located next to abandoned arsenic ore, and *P*. *vittata* is the only plant found in topsoil. Site Xiangxiong is located next to an abandoned arsenic mine, and its soil arsenic content is lower than that of Chadong. The storage capacity of arsenic-bearing ores in Lutang is relatively high. This sampling site is mainly used to stack the tailings discharged from the surrounding concentrators. Due to the low availability of arsenic ore, it is not sorted and directly discharged into the tailing pond, resulting in a high arsenic content in the tailing pond. Site Heishiding is located next to an abandoned building in the forest of a nature reserve and was used to collect control samples. The physicochemical properties of soil from the four sites were provided in Supplementary Table S1. From each site, we collected 4 samples of rhizosphere soil around *P*. *vittata*. After the *P*. *vittata* roots were removed from the soil, the soil blocks attached to the roots were gently shaken off, and then the soil attached to the roots was forcefully shaken off to obtain rhizosphere soil samples. All soil samples were shipped in iced boxes and transported back to the laboratory within 48 h. Subsamples for the molecular analysis of microbial communities were stored in −20°C refrigerator until DNA extraction. Subsamples for physicochemical analyses were air dried for 2 weeks at room temperature (ca. 25°C), and sieved through 20-mesh and 80-mesh sieves, and stored at ambient temperature until use. Four *P*. *vittata* individuals (including shoots and roots) at similar growth stages were also collected from each of the four surveyed sites. They were dried, ground into fine powders, and used for the analysis of arsenic concentrations.

*Pteris vittata* spores and *in situ* topsoil were collected from the four sampling sites for pot experiments. We planted the spores in arsenic-free seedling substrate comprised of sterilized seedling soil, vermiculite, and perlite. All plants were cultivated under a 2,000 lx light intensity, 14 h light/10 h dark period, 60% relative humidity, and 26°C/20°C day/night temperature. After culturing for 5–6 months until the plant height reached about 5 cm, we transplanted them into pots containing the corresponding *in situ* soil at each site and cultivated them in a greenhouse (12 pots filled with field soil collected from each site, 48 pots in total). Pots filled with soil recovered from each site were divided into three treatments: untreated (control), bactericide-treated, and fungicide-treated, with 4 replicates for each treatment. Bactericide consisted of ampicillin and streptomycin prepared at 100 mg L^−1^, and the fungicide Amistar was prepared at 0.2 mg ml^−1^. We watered twice a day at 6:00 AM and 7:00 PM, applied bactericide or fungicide (50 ml per pot) once a week at a fixed time, and applied Hoagland nutrient solution every 3 weeks. After 3 months of treatment, all *P*. *vittata* in pots were harvested and divided into aboveground and belowground parts, and rhizosphere soil samples were collected and processed as above mentioned.

### Determination of soil physicochemical properties

Rhizosphere soil pH and electronic conductivity (EC) were measured using a pH meter and an EC meter in a 1:2.5 (w/v) aqueous solution. Total N, nitrate-nitrogen (NO_3_^−^-N), and ammonia-nitrogen (NH_4_^+^-N) were quantified by SmartChem (SmartChem; Westco Scientific Instruments Inc., Brookfield, CT, United States) according to a previously published method (Yang et al., 2017). Total and available P were measured using the Murphy–Riley method and Olsen’s method, respectively (Olsen et al., 1954; Murphy and Riley, 1962). The total and available K were determined using standard methods (Sparks and Sparks, 1996). The soil arsenic content was determined by inductively coupled plasma-optical emission spectrometry (ICP-OES; Optima 6,300 DV, Perkin-Elmer, Wellesley, MA, United States) after HNO_3_-HClO_4_ digestion. Digested solutions with low content (<0.1 ppm) were determined by hydride generation atomic fluorescence spectrometry (HG-AFS; AFS-8220, Titan Beijing Ltd., China).

### Determination of arsenic content in plant tissue

The arsenic content in *P*. *vittata* tissue was determined by ICP-OES after sulfuric acid digestion, and samples with lower arsenic content were determined by HG-AFS. The arsenic bioaccumulation factor of the *P*. *vittata* shoot (aerial) was calculated as the ratio of the arsenic content of the aerial part to the total arsenic content of rhizosphere soil, and the arsenic bioaccumulation factor of roots was the ratio of the arsenic content of *P*. *vittata* roots to the total arsenic content of rhizosphere soil. The arsenic translocation factor was calculated as the ratio of the arsenic content in *P*. *vittata* shoots to the arsenic content in their roots. Remediation efficiency was calculated by multiplying the shoot biomass by the arsenic content in the *P*. *vittata* shoot (Ali et al., 2013).

### Molecular characterization of the rhizosphere soil microbiome

The total DNA of *P*. *vittata* rhizosphere soil was extracted using a FastDNA Spin kit for soil (MP Biomedicals, Santa Ana, CA, United States), and the DNA content was quantified using a NanoDrop 2000 spectrophotometer (Thermo Scientific, Waltham, MA, United States) to ensure the quality of DNA extraction. Universal primer pairs 515F and 806R (Bates et al., 2011) were used to amplify the V4 region of the bacterial 16S rRNA genes. Based on a nested PCR strategy (Davey et al., 2012), the fungal internal transcribed spacer 2 (ITS2) region was amplified using the primer pairs ITS1F and ITS4R. Error-correcting sample-specific barcodes were added to the forward primers 515F and ITS1F to distinguish the samples. Each DNA sample was amplified in triplicate. The PCR products were mixed equimolarly and purified using a QIAquick Gel Extraction Kit (Qiagen, Valencia, CA, United States). The composite DNA samples were subjected to 2 × 250 bp pair-end sequencing reactions on the MiSeq NextGen platform (Illumina, San Diego, CA, United States).

### Sequencing data processing and analysis of arsenic cycling genes with AsChip

Quality filtering of raw sequencing data was performed by R package *dada2*. To assign taxonomy to the sequence variants generated from the *dada2* pipeline, we used the SILVA database (release 138.1; Quast et al., 2012) and UNITE (v. 8 release; Kõljalg et al., 2013) with a confidence interval of 80%. Prediction of bacterial metabolic and other ecologically relevant functions was performed using the Python script *collapse_table*.*py* based on the FAPROTAX database (Louca et al., 2016). The functional guild assignment of fungal ASVs was implemented using the online version of FUNGuild (Nguyen et al., 2016). We also analyzed rhizosphere soil DNA using AsChip, a novel high-throughput qPCR chip, for the comprehensive quantitative profiling of genes involved in microbial arsenic cycling (Zhao et al., 2018). A total of 19 targeted genes of relevance for the microbial cycling of arsenic encoding As(III) oxidation, As(V) reduction, arsenic transport, and arsenic methylation/demethylation were quantified.

### Indicator species and network analysis

We employed correlation-based indicator species analysis with the R package *indicspecies* to calculate the correlation coefficient of an amplicon sequence variant (ASV)’s positive association with one or more arsenic contamination levels. ASVs whose relative abundances were identified as differing between one or more arsenic contamination levels at a false discovery rate corrected value of *p* < 0.05 were considered to be arsenic contamination level responsive. We then selected the ASVs that were both abundant (top 10% of all identified bacterial/fungal ASVs in terms of relative abundance) and ubiquitous (>25% of all samples) to build a co-occurrence network under each arsenic contamination level. Pairwise correlation coefficients were calculated to quantify the biological interactions among ASVs detected in the rhizosphere soil of *P*. *vittata*, while *p*-values were adjusted by the Benjamini and Hochberg linear step-up procedure (Benjamini et al., 2006) to control the false discovery rate. Valid linkages were defined using a threshold of Spearman’s |*ρ*| ≥ 0.65 and false discovery rate-adjusted *p* < 0.001. Bipartite networks and co-occurrence networks were constructed using an R package *igraph* (Csardi and Nepusz, 2006) and visualized by Gephi 0.9.2 (Bastian et al., 2009).

### Statistical analysis

To illustrate the effects of soil arsenic content on microbial diversity, we analyzed the relationships between soil arsenic content and bacterial and fungal richness using univariate linear regression. We quantified the effect of arsenic contamination level in the rhizosphere soil of *P*. *vittata* by constrained analysis of principal coordinates (CAP) based on the Bray–Curtis distance. To explore the relationships between soil arsenic content and the relative abundance of bacterial and fungal genera, predicted bacterial functions, and fungal functional groups, we calculated Spearman’s correlation coefficients between these variables. The Duncan test was used to test significant differences among arsenic contamination levels in terms of arsenic remediation efficiency and other arsenic-related properties of *P*. *vittata*.

## Results

### Microbial diversity and other environmental factors

**Figure 1:**
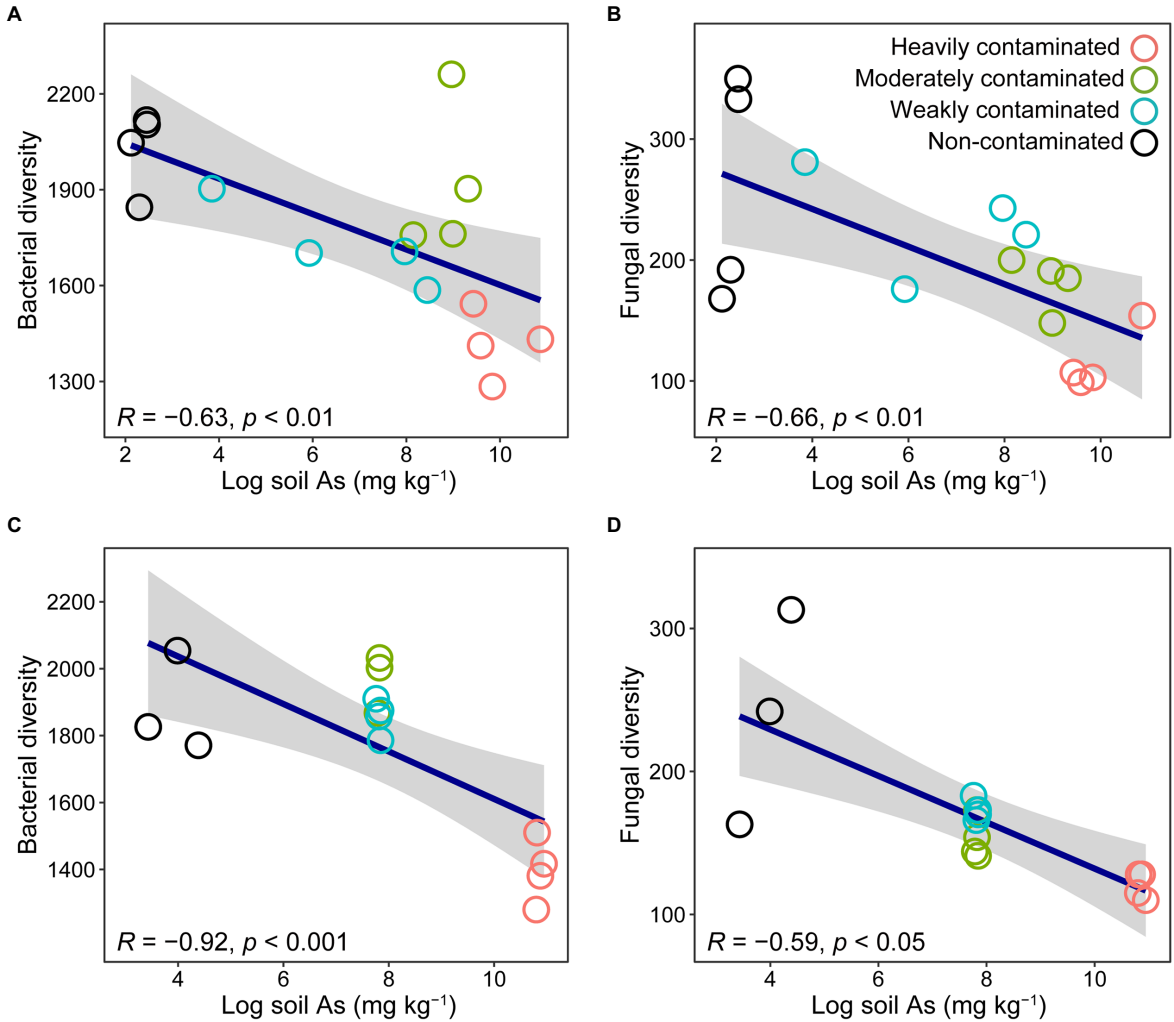
Relationship between soil arsenic content and bacterial **(A,C)** or fungal **(B,D)** richness in rhizosphere soil of *Pteris vittata* sampled in field **(A,B)** and pot experiments **(C,D)**. Open circles in different colors represent soil samples with different arsenic contamination levels. The gray shaded areas around the regression lines indicate the 95% confidence interval.

Among the sites we surveyed representing four levels of arsenic contamination, bacterial richness in the rhizosphere soil of *P*. *vittata* was significantly negatively correlated with soil arsenic content (Figure 1A). The number of bacterial ASVs in heavily contaminated soil (1,417 ± 106), moderately contaminated soil (1921 ± 236), and weakly contaminated soil (1724 ± 113) were significantly lower than that in non-contaminated soil (2028 ± 126, *p* < 0.05). We found similar patterns when we conducted pot experiments with *in situ* soil collected from each site. In the pot experiment, bacterial richness was also significantly negatively correlated with soil arsenic content (Figure 1C).

In the four sites investigated in the field, the rhizosphere soil fungal richness of *P*. *vittata* was significantly negatively correlated with soil arsenic content (Figure 1B). The number of fungal ASVs in heavily contaminated soil (115 ± 25), moderately contaminated soil (181 ± 22), and weakly contaminated soil (230 ± 43) were significantly lower than that in non-contaminated soil (260 ± 94, *p* < 0.05). Similarly, in the pot experiment, the diversity of rhizosphere soil fungi was also significantly negatively correlated with soil arsenic content (Figure 1D).

The arsenic translocation factors of *P*. *vittata* and the arsenic bioaccumulation factor of the belowground parts of *P*. *vittata* did not change significantly among sites at different contamination levels (Supplementary Figures S2A,E). However, the arsenic content in both the aboveground and belowground tissues of *P*. *vittata* decreased with a decrease in arsenic content in the soil (Supplementary Figures S2B,D). The arsenic bioaccumulation factor of the aboveground part of *P*. *vittata* was significantly higher at the non-contaminated site than in the heavily contaminated and moderately contaminated sites (Supplementary Figure S2C). Arsenic contamination in soil can also affect other environmental factors. Specifically, soil nutrient elements, such as total nitrogen and total phosphorus content in the aboveground and belowground tissues of *P*. *vittata*, were significantly negatively correlated with arsenic content in the soil (Supplementary Figure S3).

### Response of microbial community composition to soil arsenic contamination

**Figure 2:**
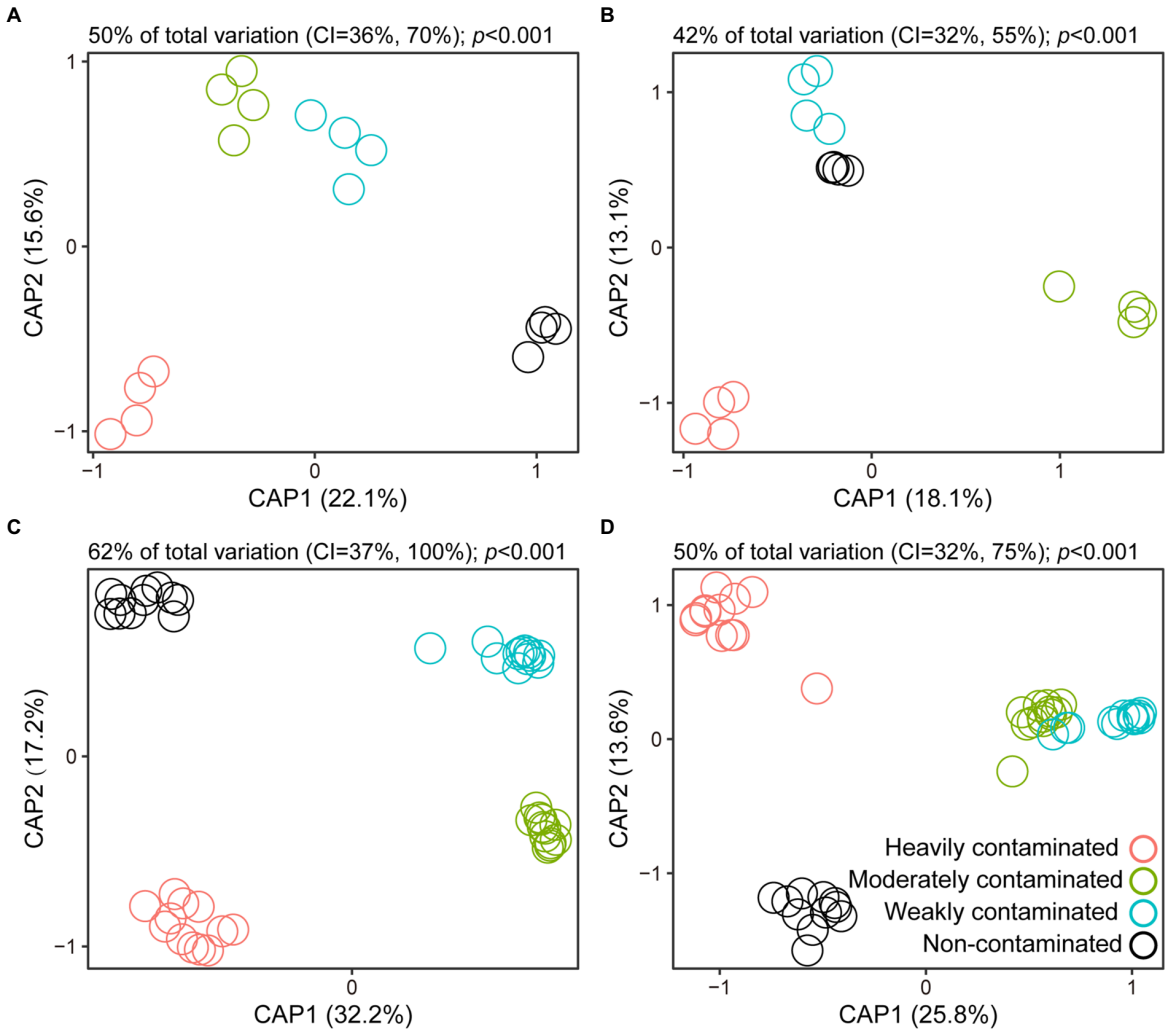
Canonical analysis of principal coordinates (CAP) based on Bray–Curtis distances for visual presentations of differences in bacterial and fungal community composition in rhizosphere soil of *Pteris vittata* sampled in field **(A,B)** and pot **(C,D)** experiments. Open circles in different colors represent soil samples with different arsenic contamination levels.

The results of CAP analysis based on field survey data showed that there were significant differences in the bacterial and fungal community composition in the rhizosphere soil of *P*. *vittata* (Figures 2A,B). Similarly, the response of soil bacterial and fungal community composition to soil arsenic content in the pot experiment was consistent with the results of the field experiment (Figures 2C,D). This indicates that soil arsenic contamination has an important effect on the community structure of the rhizosphere microorganisms of *P*. *vittata.*

**Figure 3:**
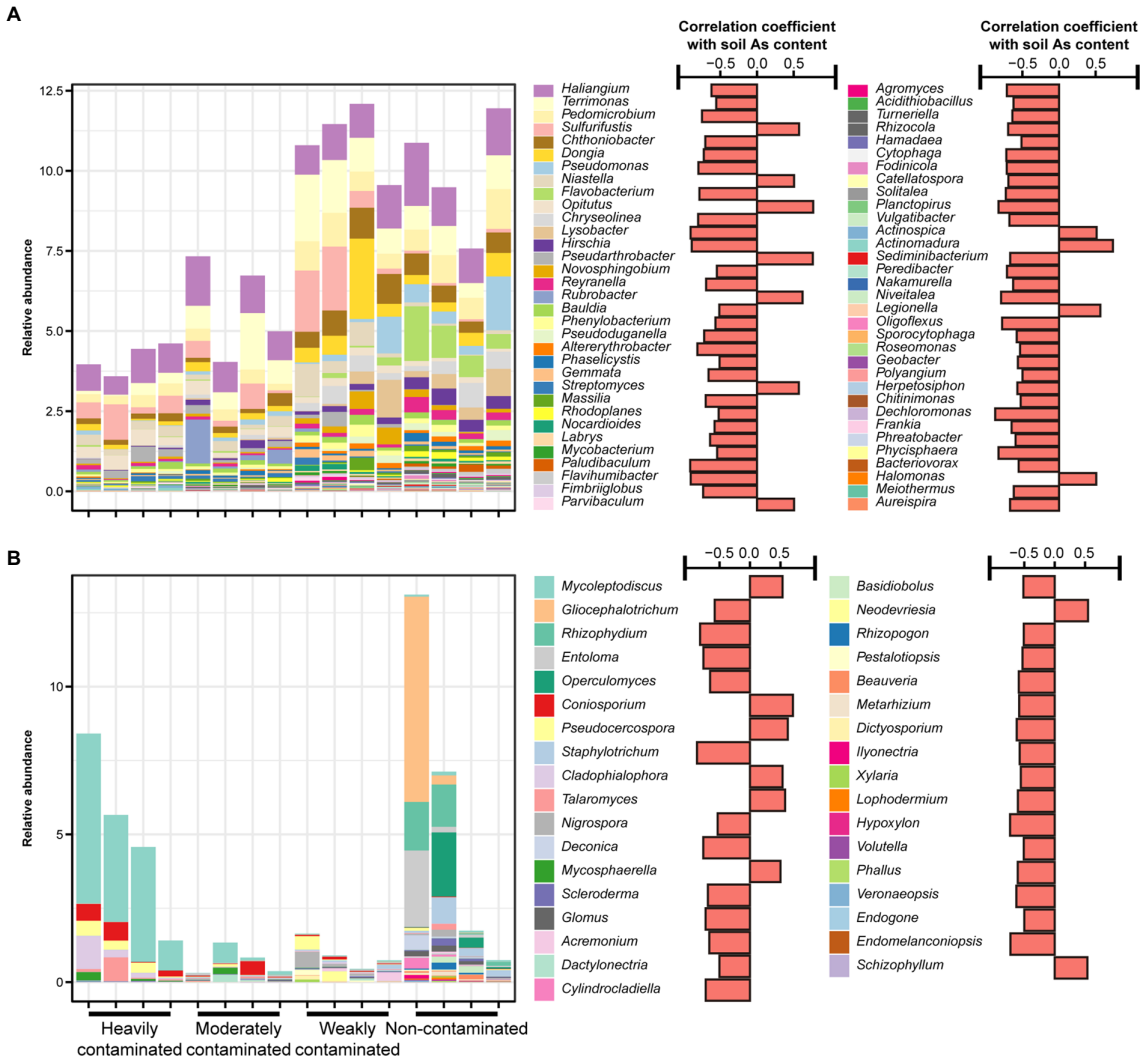
Relative abundances of **(A)** bacterial and **(B)** fungal genera that significantly correlate with arsenic content in *Pteris vittata* rhizosphere soil in the field. The bar plots on the right show the correlation coefficients between the relative abundance of these genera and soil arsenic content.

In addition, we further analyzed the responses of the relative abundance of dominant bacterial and fungal taxa in the rhizosphere soil of *P*. *vittata* to soil arsenic contamination. From the bacterial phylum level, the most dominant phylum in the soils of the four contamination levels was Proteobacteria. The relative abundance of Bacteroidetes was significantly negatively correlated with soil arsenic content (*R* = −0.50, *p* < 0.05, Supplementary Figure S4A). At the genus level, *Sulfurifustis* (*R* = 0.57, *p* < 0.05), *Niastella* (*R* = 0.51, *p* < 0.05), *Opitutus* (*R* = 0.77, *p* < 0.01), and *Pseudarthrobacter* (*R* = 0.76, *p* < 0.01) showed significant positive correlations with soil arsenic content (Figure 3A). Dominant genera *Haliangium* (*R* = −0.62, *p* < 0.05), *Terrimonas* (*R* = −0.56, *p* < 0.05), *Pedomicrobium* (*R* = −0.75, *p* < 0.01), *Chthoniobacter* (*R* = −0.71, *p* < 0.01), *Dongia* (*R* = −0.73, *p* < 0.01), and *Bauldia* (*R* = −0.51, *p* < 0.05) were negatively correlated with soil arsenic content. The relative abundance of *Flavobacterium* (*R* = −0.79, *p* < 0.01) was significantly affected by soil arsenic contamination, and the relative abundance in heavily contaminated sites was 0.019%, while in weakly contaminated sites and non-contaminated sites, the relative abundances were 0.12% and 0.96%, respectively (Figure 3A).

For soil fungi, Ascomycota was the most dominant phylum, but at the phylum level, there was no significant correlation between fungal phylum and arsenic content in the rhizosphere soil of *P*. *vittata* (Supplementary Figure S4B). At the genus level, the dominant fungi in the soils of the four contamination levels were significantly different. The dominant genera in heavily and moderately contaminated sites were *Mycoleptodiscus* and *Coniosporium*, and the dominant genera in weakly contaminated sites were *Pseudocercospora* and *Nigrospora*. In the non-contaminated sites, the dominant genera were *Gliocephalotrichum*, *Rhizophydium*, *Entoloma*, and *Operculomyces*. In Ascomycota, *Mycoleptodiscus* (*R* = 0.54, *p* < 0.05), *Talaromyces* (*R* = 0.58, *p* < 0.05), *Cladophialophora* (*R* = 0.53, *p* < 0.05), and *Mycosphaerella* (*R* = 0.50, *p* < 0.05) were positively correlated with soil arsenic content, and *Schizophyllum* belonging to Basidiomycota (*R* = 0.78, *p* < 0.01) was also positively correlated with soil arsenic content. Arbuscular mycorrhizal fungi *Glomus* (*R* = −0.73, *p* < 0.01) was negatively correlated with soil arsenic content.

To explore which microbes were indicator taxa for a particular arsenic contamination level, we employed indicator species analysis to identify individual ASVs in soil microbial communities whose relative abundance varied between contamination levels (Supplementary Figure S5). Consistent with the finding that contamination level explains variation among soil bacterial and fungal communities (Figure 2), we identified indicator ASVs specific to each contamination level. There was little overlap between contamination levels for both bacteria and fungi. Specifically, the number of bacterial indicator species for soils with high, moderate, low, and no contamination levels was 77, 70, 56, and 203, respectively, while fungal indicator species from heavily contaminated to non-contaminated soils were 9, 15, 15, and 34 (Supplementary Figure S5). No species were indicative of soils at all four levels of contamination in either the bacterial or fungal datasets. However, two bacterial ASVs belonging to *Actinobacteria* and *Verrucomicrobia* were indicator species between heavily contaminated and non-contaminated soils, and one fungal ASV belonging to Ascomycota was an indicator species between moderately contaminated and non-contaminated soils (Supplementary Figure S5). Taken together, each contamination level supported a specialized subset of soil bacteria and fungi, while very few microbes were shared between the sampling sites.

Co-occurrence network analysis of rhizosphere soil microorganisms showed that the modularity and number of nodes decreased with an increase in arsenic content. The interactions between microorganisms in arsenic-contaminated soils were stronger than those in non-contaminated soils (Supplementary Figure S6), suggesting that an increase in arsenic content could increase the interaction between microorganisms. Interestingly, in arsenic-contaminated soil, as the arsenic content decreased, bacteria–bacteria and bacteria–fungi interactions became more frequent, and fungi–fungi links were stronger in environments with a higher arsenic content (Supplementary Figure S6), indicating that the effects of increasing arsenic content on the interactions between interkingdom and intrakingdom microbes were different.

### Response of predicted bacterial functions and fungal functional guilds to soil arsenic contamination

**Figure 4:**
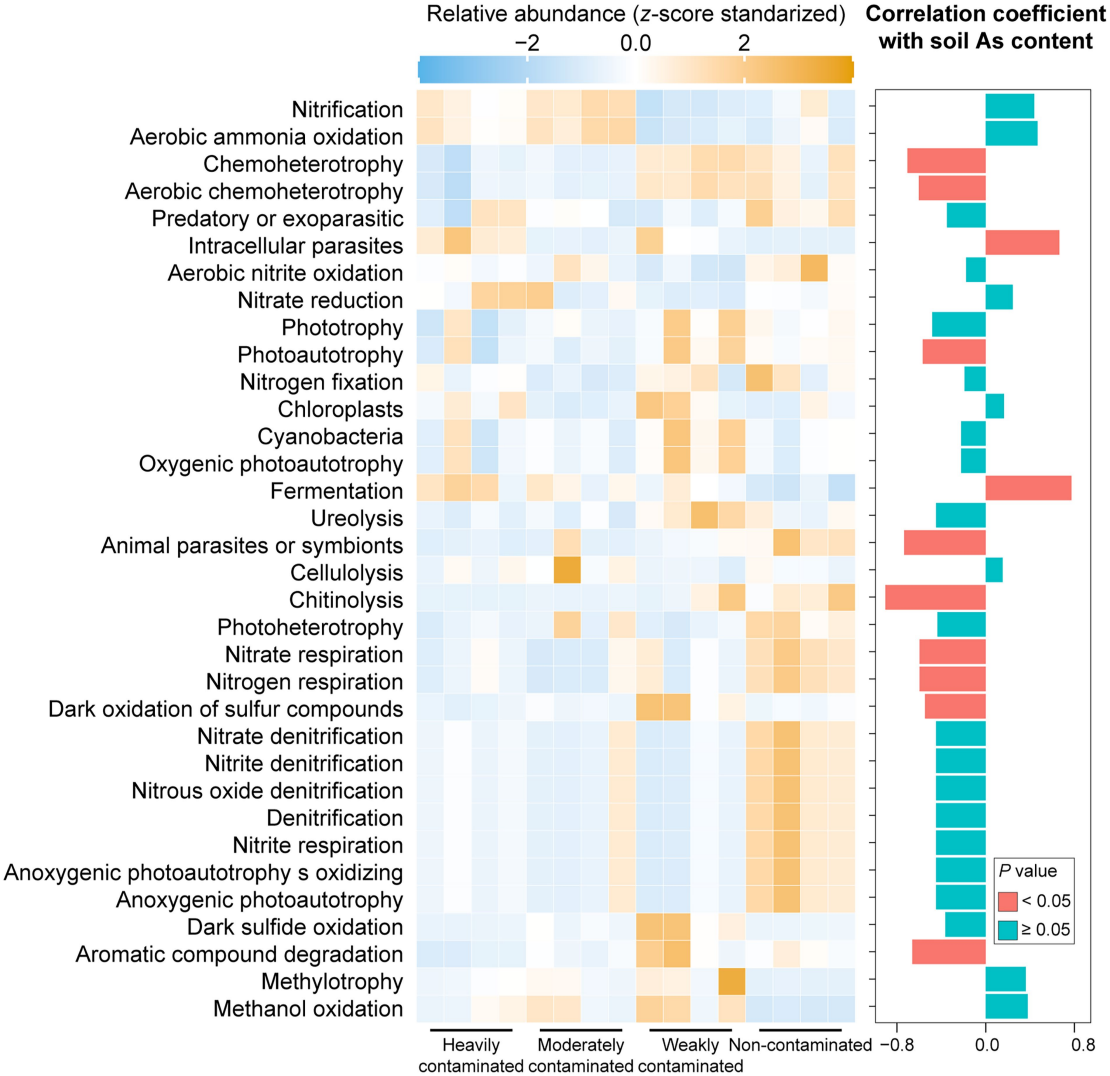
Relative abundance of predicted bacterial functions based on the FAPROTAX database in rhizosphere soil of *Pteris vittata* with different levels of arsenic contamination in the field. Colors in the heatmap represent the relative abundance of predicted bacterial functions in *P*. *vittata* rhizosphere soil with different arsenic contamination levels in the field. The bar plot on the right shows the correlation coefficients between predicted bacterial functions and soil arsenic content. The colors of the bars indicate the significance of the correlations between predicted functions and soil arsenic content.

The putative metabolic and ecologically relevant functions of bacterial ASVs predicted by the FAPROTAX database showed that functional groups linked to the biogeochemical cycling of carbon (e.g., chitinolysis), nitrogen (e.g., nitrate respiration, and nitrogen respiration), sulfur (e.g., dark oxidation of sulfur compounds), and trophic types (e.g., chemoheterotrophy, photoautotrophy, and aerobic chemoheterotrophy) were negatively correlated with soil arsenic content (Figure 4). Carbon cycling functional groups (e.g., fermentation) and functional groups associated with intracellular parasites were positively correlated with soil arsenic content (Figure 4), suggesting that increased arsenic contamination in the soil environment may enhance the damage of parasitic bacteria to plants.

**Figure 5:**
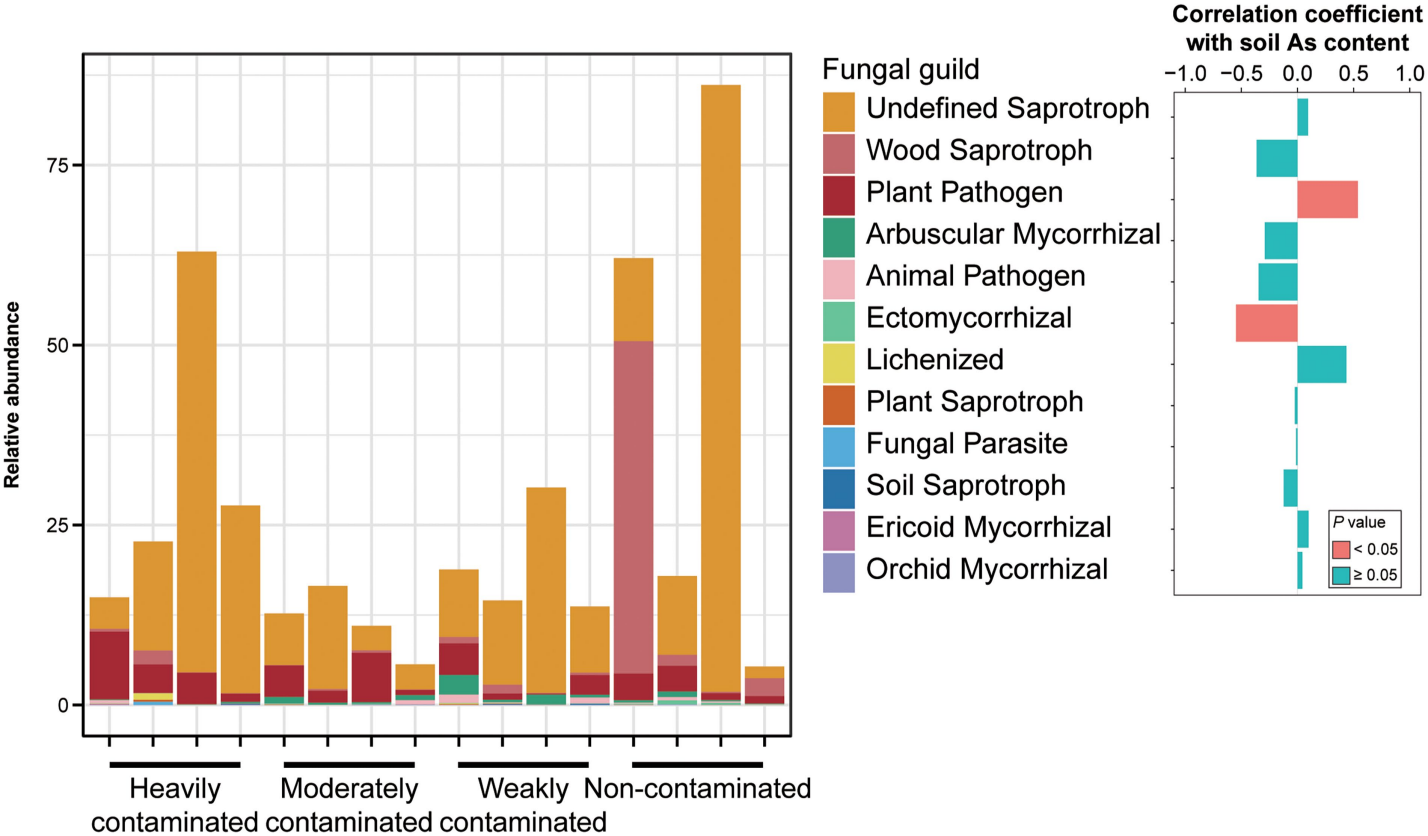
Relative abundances of fungal functional groups in *Pteris vittata* rhizosphere soil with different levels of arsenic contamination in the field. The bar plot on the right shows the correlation coefficients between these fungal guilds and soil arsenic content. The colors of the bars indicate the significance of the correlations between the relative abundance of soil fungal guilds and soil arsenic content (red: *p* < 0.05, green, *p* > 0.05).

For different functional guilds of soil fungi, the relative abundance of plant fungal pathogens showed a significantly positive correlation with soil arsenic content, while the relative abundance of ectomycorrhizal fungi showed a significantly negative correlation (Figure 5). The relative abundance of arbuscular mycorrhizal fungi was negatively correlated, but not significantly, with soil arsenic content. These results suggest that the aggravation of arsenic contamination not only increased the abundance of pathogenic fungi in the rhizosphere soil but also significantly reduced the abundance of symbiotic fungi.

### Response of arsenic-cycling genes to soil arsenic contamination

**Figure 6:**
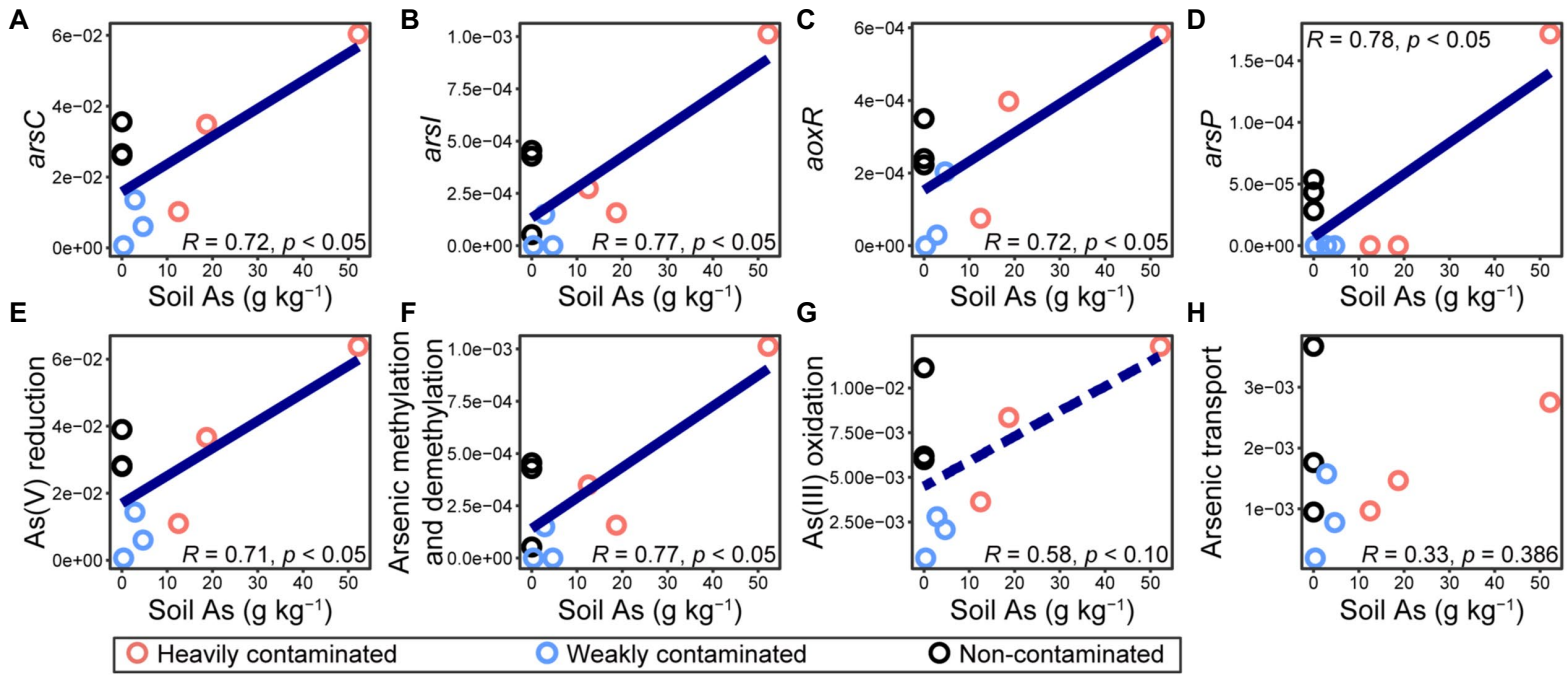
Normalized abundances (i.e., arsenic gene copy number per 16S rRNA gene) of **(A–D)** genes involved in microbial arsenic cycling and **(E–H)** different functional processes in *Pteris vittata* rhizosphere soil with different levels of arsenic contamination in the field. The abundance of **(A)** *arsC*, **(B)** *arsI*, **(C)** *aoxR*, and **(D)** *arsP*, which are involved in As(V) reduction, arsenic methylation, As(III) oxidation, and arsenic transport, respectively, were analyzed with a high-throughput qPCR chip (AsChip). The abundance of functional processes refers to the sum of abundances of all genes encoding four main arsenic biotransformation processes (i.e., arsenic methylation and demethylation, arsenic transport, As(III) oxidation, and As(V) reduction).

In this study, we used high-throughput qPCR AsChip to quantify soil arsenic cycling-related genes in the rhizosphere microorganisms of *P*. *vittata*. The abundance (normalized, %) of genes detected by AsChip and their corresponding functional processes were shown in Supplementary Table S2. With the increase of arsenic contamination in the soil environment, the abundance of genes encoding arsenic reduction, arsenic methylation, and demethylation increased significantly (Figures 6E,H). The abundance of *arsC*, *arsI*, *aoxR*, and *arsP*, which are involved in As(V) reduction, arsenic methylation, As(III) oxidation, and arsenic transport, respectively, increased significantly with the increase in soil arsenic content (Figures 6A–D). However, none of these gene types, whose abundances were significantly negatively correlated with soil arsenic content, was detected in our dataset. The increase in arsenic contamination level led to the accumulation of microorganisms with genes encoding arsenate resistance by the arsenate reduction-arsenite efflux pathway, demethylation of trivalent organoarsenicals to less toxic As(III), and arsenic methylation.

### Regulatory effects of bactericide and fungicide on soil microbial community composition and phytoremediation efficiency

**Figure 7:**
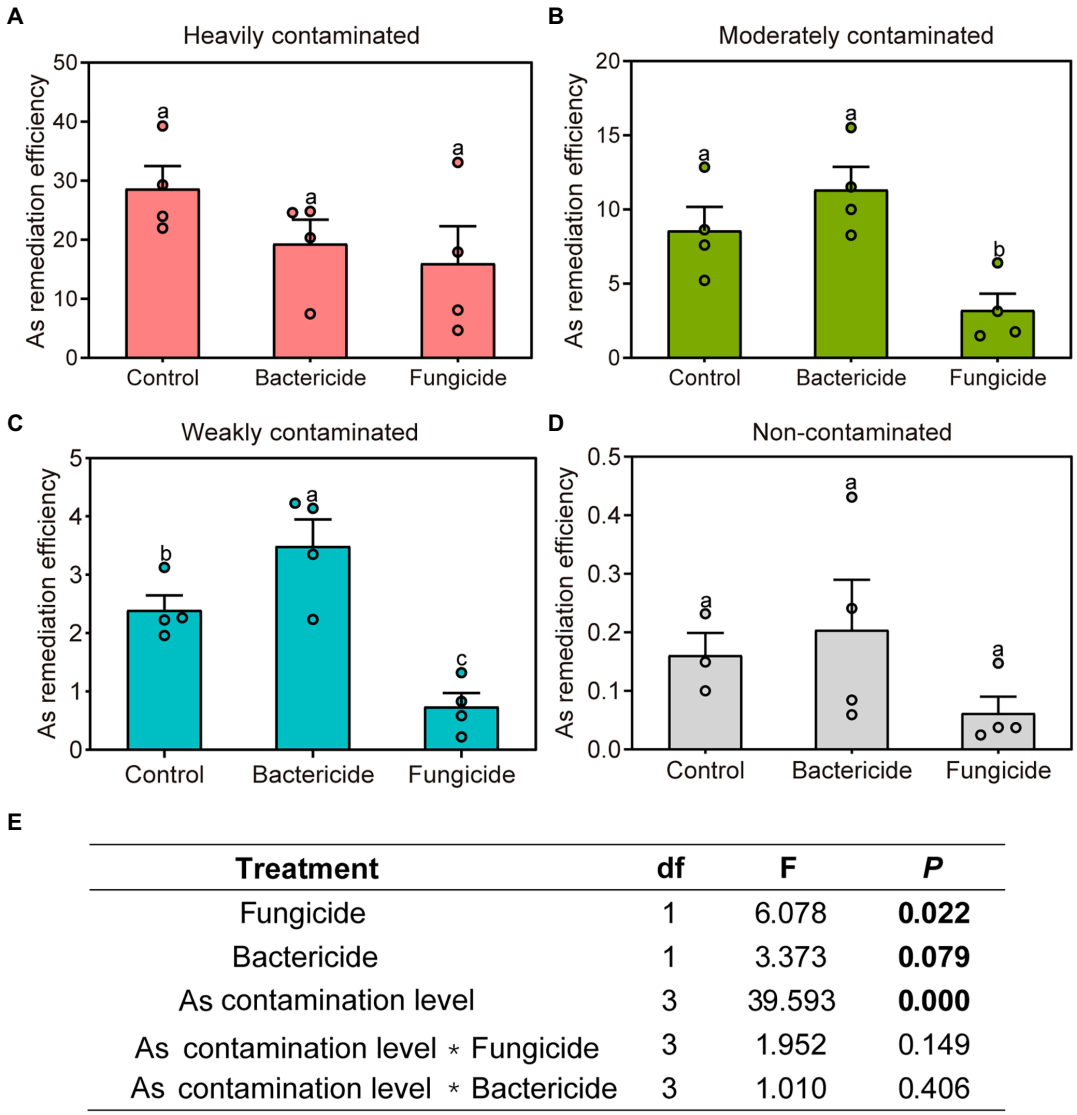
Effects of bactericide or fungicide application on the remediation efficiency of *Pteris vittata* grown in sites with different levels of arsenic contamination reflected by **(A–D)** Duncan multiple comparison and **(E)** analysis of variance. Different lowercase letters on bar plots denote significant differences between treatments (Duncan multiple comparison, *p* < 0.05).

After the application of bactericides, the overall remediation efficiency of *P*. *vittata* changed marginally insignificantly when grown under soils collected from an arsenic contamination gradient (*p* = 0.079, Figure 7E). In weakly contaminated soil (Figure 7C), the application of bactericides significantly improved the remediation efficiency of *P*. *vittata*, but such an effect did not appear in other arsenic-contaminated sites. The application of fungicides significantly reduced the remediation efficiency of *P*. *vittata* overall (*p* = 0.022, Figure 7E), and this effect was particularly significant in moderately and weakly contaminated soils (Figures 7B,C) but not significant in heavily contaminated and non-contaminated sites (Figures 7A,D). Therefore, the role of microorganisms, especially fungi, was particularly important in the process of remediation of arsenic-contaminated soil by *P*. *vittata*.

Furthermore, bacterial richness was significantly negatively correlated with arsenic content in *P*. *vittata* roots after bactericide and fungicide treatment (Supplementary Figure S7A), but the change in bacterial community composition was not significant (Supplementary Figure S8A). Spraying bactericide and fungicide mainly affected genera belonging to Proteobacteria, Acidobacteria, and Actinobacteria (Supplementary Table S3) and also affected some nutrient metabolic functions of bacteria, such as nitrite denitrification and photoautotrophy (Supplementary Tables S4, S5).

The relationship between fungal richness and arsenic content in *P*. *vittata* roots was not affected by bactericide and fungicide treatments and still showed a significant negative correlation (Supplementary Figure S7B), but the changes in fungal community composition were not significant (Supplementary Figure S8C). The application of bactericide and fungicide to the pots mainly affected some genera attributable to Ascomycota in the fungal community, such as *Myrothecium* (Supplementary Table S6), and affected fungal functional guilds, such as arbuscular mycorrhizal fungi and saprotrophic fungi (Supplementary Table S7).

## Discussion

### Effect and potential mechanism of arsenic pollution on bacterial and fungal diversity and community structure

Our *in situ* investigation and microcosm experiments showed that soil arsenic pollution reduced the bacterial and fungal diversity in the rhizosphere of *P*. *vittata* and is of vital importance in shaping the community structure of bacteria and fungi, indicating the significant role of soil arsenic pollution in driving the microbial environment of *P*. *vittata*. The first hypothesis of this study, i.e., the soil arsenic level plays an important role in shaping the microbial community structure, was thus supported. Das et al. (2017) and Yu et al. (2020) also demonstrated that soil arsenic pollution significantly reduced bacterial diversity in the *P. vittata* rhizosphere. Many studies concerning the effects of arsenic on microbial diversity in the rhizosphere of *P*. *vittata* have focused on culturable bacterial and fungal groups (Turpeinen et al., 2004; Das et al., 2016, 2017; Xiao et al., 2021). These studies have usually reported a reduced diversity of culturable bacteria and fungi due to arsenic pollution (Das et al., 2016), and they have also shown that arsenic pollution leads to an increase in the relative abundance of bacteria and fungi species/strains with tolerance and/or metabolic functions for arsenic (Turpeinen et al., 2004; Das et al., 2017). Additionally, in our work, we observed the enrichment of several As-related microbes, including *Sulfurifustis*, which are tolerant to arsenic (Sun et al., 2020), and *Opitutu*, which are involved in the transport and flow of soil arsenic (Giloteaux et al., 2013).

There are several explanations for the strong effect of soil arsenic pollution on bacterial and fungal communities. First, arsenic toxicity can directly inhibit the growth and reproduction of arsenic-sensitive bacteria and fungi (Nannipieri et al., 2012; Das et al., 2013). Among the microbial groups co-related with arsenic pollution, most groups were negatively correlated with soil arsenic concentration (Figure 3; Supplementary Figure S4), implying the inhibitory effect of arsenic toxicity on a considerable number of bacterial and fungal groups. Second, arsenic pollution may increase the amount of root exudates from *P*. *vittata* (Das et al., 2017), which may enhance the selective effect of plants on bacterial and fungal communities through the “rhizosphere effect,” thereby reducing microbial diversity in the rhizosphere. Third, soil arsenic pollution significantly reduced plant P concentration (Supplementary Figure S3), possibly by competing with P for the phosphate transporter in *P*. *vittata* roots (Wang et al., 2002; Wu et al., 2022b), which is likely to further impact the microbial community in the rhizosphere of *P*. *vittata*.

### Effects of soil arsenic pollution on the functional role of rhizosphere microbial groups

In this work, soil arsenic pollution reduced the suitability of soil microbes for the growth of *P*. *vittata*; it significantly increased the relative abundance of parasitic bacteria and pathogenic fungi while decreasing the abundance of symbiotic fungi, such as ECM and AMF. The second hypothesis of this study, i.e., the effects of arsenic on the microbial community further impact the arsenic accumulation ability of *P*. *vittata*, was, to some degree, also supported. There are several possible explanations for these results. First, soil arsenic pollution significantly hindered the absorption of P by *P*. *vittata* (Supplementary Figure S3), possibly due to competition between arsenic and P for the phosphate transporter in *P*. *vittata* roots (Wu et al., 2022b). Laliberté et al. (2015) found a trade-off for plants in soil P acquisition and resistance to soil-borne pathogens. The hindrance of arsenic pollution on P acquisition may have reduced the pathogenic resistance of *P*. *vittata*. Second, for most plants, toxic heavy metals at a low dose may have beneficial effects, termed hormesis, and at a high dose, they have harmful effects (Morkunas et al., 2018). Therefore, the deleterious effects of toxic arsenic on the immune system of *P*. *vittata* may also contribute to the enrichment of pathogenic fungi and parasitic bacteria following increased arsenic pollution. Third, some plant pathogens (e.g., *Fusarium oxysporum*) have also acquired the ability to adapt to heavy metals during their co-evolution with the immune system of plants (Lorenzo-Gutiérrez et al., 2019; Bentham et al., 2021). The higher adaptability to heavy metals (e.g., As) may help pathogenic fungi gain an advantage in competition with symbiotic fungi and other microbial groups under arsenic pollution. Our results emphasize the significant role of heavy metals in the soil microbial community and suggest that the regulation of soil microorganisms is a promising way for successful phytoremediation in heavy metal-polluted environments.

Soil arsenic pollution significantly increased the abundance of functional genes of As(III) oxidation, As(V) reduction, and arsenic methylation and demethylation (Figure 6), indicating the enrichment effect of arsenic pollution on arsenic functional genes (microorganisms) in the rhizosphere of *P*. *vittata*. Previous studies have shown that *P*. *vittata* and other plants are more tolerant of As(V) than As(III), and their uptake of soil arsenic is mainly in the form of As(V) (Yan et al., 2019; Yang et al., 2022). In this work, the enrichment of functional genes in arsenic transformation in arsenic-polluted soil should also impact the phytoremediation of soil arsenic by *P*. *vittata*. The enrichment of As(III) oxidation functional genes in arsenic-polluted soil, including the *aoxR* genes encoding the key protein of bacterial As(III) oxidation (Kostal et al., 2004), can not only reduce the toxicity of arsenic to plants but also improve the remediation efficiency of *P*. *vittata*. In this study, soil arsenic pollution also led to the enrichment of As(V)-reducing genes (e.g., ArsC), which may hinder plant growth and arsenic uptake, thereby reducing phytoremediation efficiency. Ghosh et al. (2011) surveyed the relationship between soil bacteria and arsenic forms in soil and found that some arsenic-tolerant bacteria transformed the forms of soil arsenic to improve their fitness (Ghosh et al., 2015). Here, the enrichment of As(V)-reducing genes (microorganisms) by arsenic pollution may reflect the adaptability of rhizosphere soil microorganisms to arsenic pollution, since for most microorganisms the cytotoxicity of As(V) is higher than that of As(III) (Ghosh et al., 2015). Overall, our results on arsenic-related functional genes further confirm the important role and great application potential of soil microorganisms in the phytoremediation of arsenic pollution in the natural environment.

### Effects of indigenous microorganisms on arsenic remediation efficiency

The regulation of indigenous soil microbes by the field application of fungicide, but not bactericide, significantly reduced the remediation efficiency of *P*. *vittata* grown in sites with different arsenic levels, indicating the important role of indigenous microbial groups, especially fungal groups, in the remediation of arsenic-contaminated soil, further supporting the second hypothesis of this study. By inoculating with bacterial (Xiao et al., 2020) and fungal (such as AMF, Cantamessa et al., 2020) strains isolated from the rhizosphere, previous studies have reported improvement of phytoremediation in *P*. *vittata* (Zhang et al., 2020; Mitra et al., 2022). However, most of these studies were conducted in a controlled environment (e.g., pot experiment), and there were few successful cases of *in situ* inoculation of microorganisms to improve the remediation efficiency of *P*. *vittata* under field conditions (Yang et al., 2022). The difficulty of target microorganisms surviving and adapting to field environments is an important reason for the poor effect of field inoculation tests (Wan et al., 2009). The results of this study show that in field environments, the interaction between *P*. *vittata* and indigenous microorganisms, especially fungi, in arsenic-polluted environments is very important for maintaining plant growth and supporting arsenic remediation. The interruption of this plant–microbe interaction, for instance, the use of fungicides or inoculation with certain microorganisms, may reduce the growth of *P*. *vittata* and thereby the remediation efficiency of soil arsenic pollution. Previous studies have reported that AMF can improve the phytoremediation efficiency of *P*. *vittata* by promoting plant growth (Ghosh et al., 2011) and/or enhancing the arsenic absorption ability of plant roots (Yang et al., 2012; Cantamessa et al., 2020). In this study, the reduction in the remediation efficiency of *P*. *vittata* to arsenic pollution upon fungicide application may be partly attributed to the suppression of the symbiotic relationship between AMF and *P*. *vittata*, as suggested by the lower abundance of AMF in soil with fungicide treatment (Supplementary Table S2).

## Conclusion

In summary, arsenic pollution played a significant role in shaping the microbial community structure and function in the rhizosphere of *P*. *vittata*, leading to reduced suitability of soil microbes for the growth of *P*. *vittata* and the enrichment of microorganisms carrying arsenic metabolism genes in soil, both of which should influence the phytoremediation efficiency of plants. The regulation of indigenous soil microbes by the field application of fungicide further supported the important role of indigenous microbial groups in the remediation of arsenic-contaminated soil. Microbiome engineering is a rapidly evolving frontier for solutions to improve ecological restoration (Albright et al., 2022). Our work provides novel insights into the application prospects of soil microorganisms in the phytoremediation of arsenic-polluted soil.

## Data availability statement

The data presented in the study are deposited in the EUROPEAN GENOME-PHENOME ARCHIVE repository accession number PRJEB55130.

## Author contributions

J-tL and YW planned and designed the research. PJ, FL, YW, SZ, and GW performed the experiments and analyzed the data. PJ, YW, and FL wrote the manuscript. J-tL and YW supervised the project. All authors contributed to the article and approved the submitted version.

## Funding

This study was financially supported by the National Natural Science Foundation of China (nos. 41622106, 31772397, 42177009, and 42077117), the Key-Area Research and Development Program of Guangdong Province (no. 2019B110207001), the Guangdong Basic and Applied Basic Research Foundation (no. 2021B1515120039), the Natural Science Foundation of Guangdong Province of China (nos. 2020A1515010937 and 2020A1515110972), and the YangFan Innovative and Entrepreneurial Research Team Project (2015YT02H032).

## Conflict of interest

YW was employed by Dongli Planting and Farming Industrial Co., Ltd.

The remaining authors declare that the research was conducted in the absence of any commercial or financial relationships that could be construed as a potential conflict of interest.

## Publisher’s note

All claims expressed in this article are solely those of the authors and do not necessarily represent those of their affiliated organizations, or those of the publisher, the editors and the reviewers. Any product that may be evaluated in this article, or claim that may be made by its manufacturer, is not guaranteed or endorsed by the publisher.
