# Supplementary material for: Microbial community composition in the rhizosphere of Pteris vittata and its effects on arsenic phytoremediation under a natural arsenic contamination gradient
Source: Front Microbiol. 2022 Sep 6;13:989272. doi: 10.3389/fmicb.2022.989272 (PMC9495445; doi:10.3389/fmicb.2022.989272)
Supplement: Supplementary file 1 [file Data_Sheet_1.PDF]

Supplementary Material

Supplementary Figures (S1−S8)

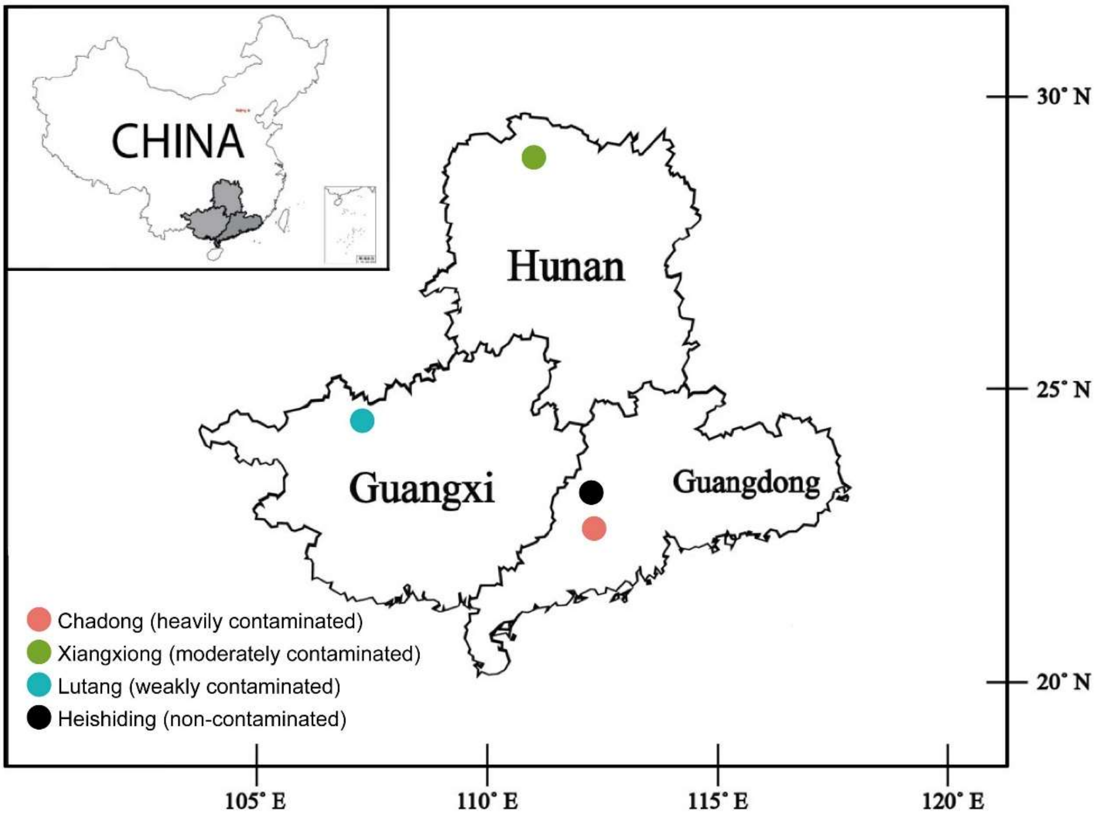

Supplementary Figure S1 | Geographic distribution of sampling sites for collecting *Pteris vittata* and the rhizosphere soil.

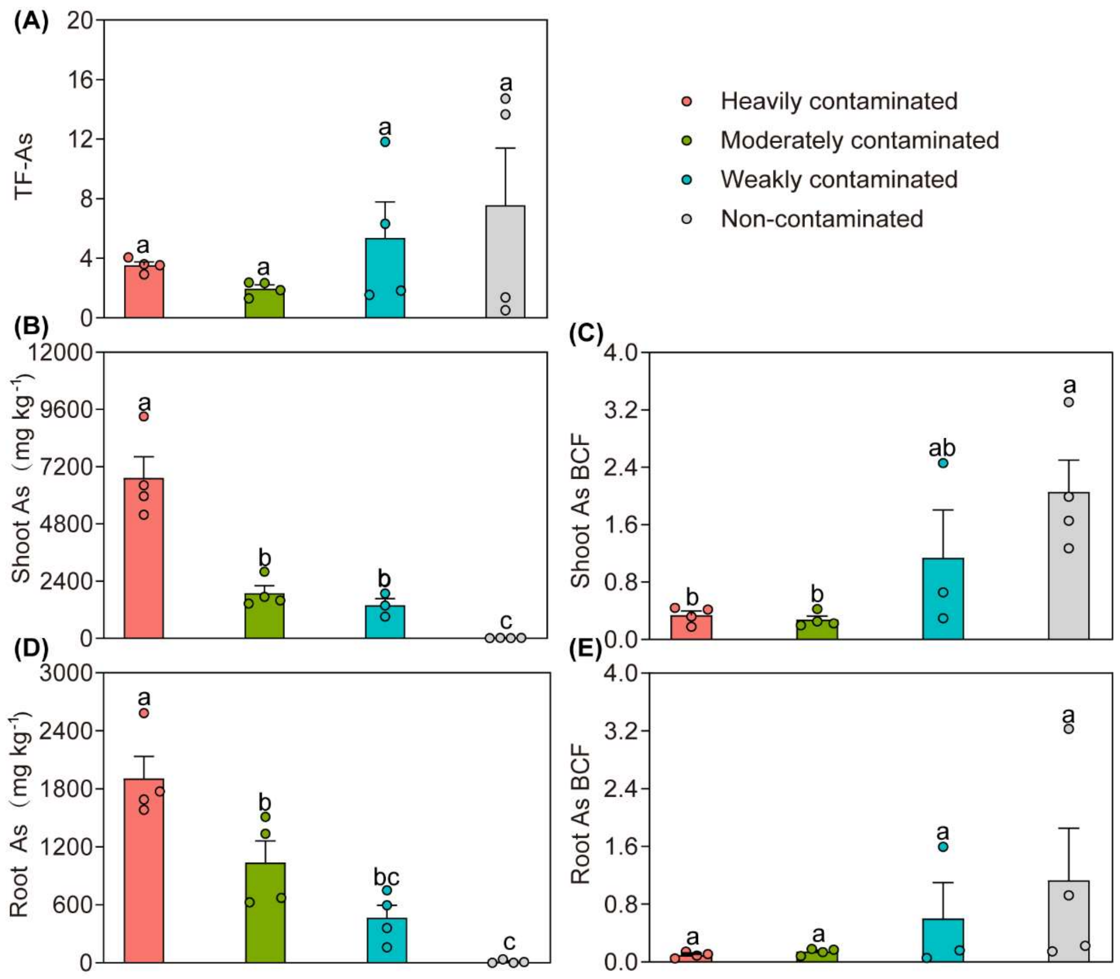

Supplementary Figure S2 | Arsenic-related parameters of *Pteris vittata* based on field sampling data. Different lowercase letters above the bars denote significant differences between treatments (Duncan multiple comparison, $p < 0.05$).

Abbreviations: TF, translocation factors; BCF, bioaccumulation factors.

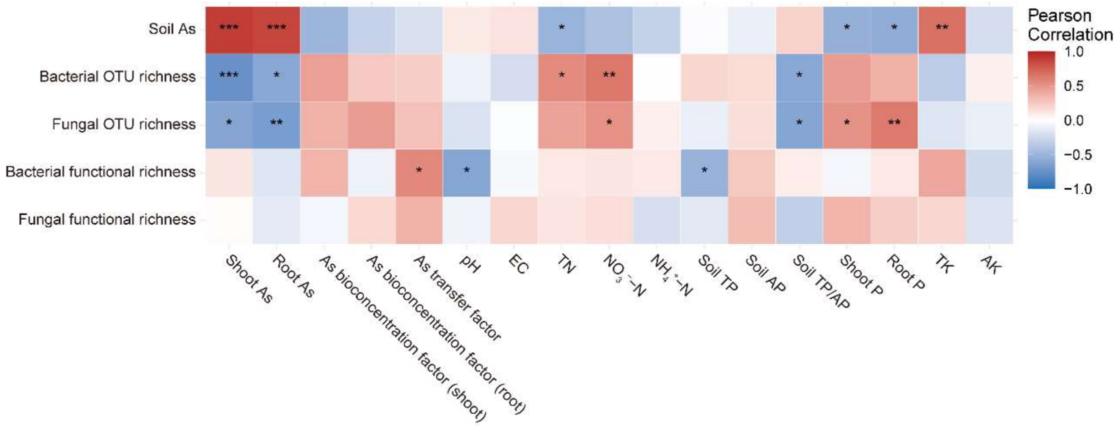

Supplementary Figure S3 | Correlation coefficients between environmental variables and microbial alpha diversity based on field sampling data.

Significance levels: *, $p < 0.05$; **, $p < 0.01$; ***, $p < 0.001$.

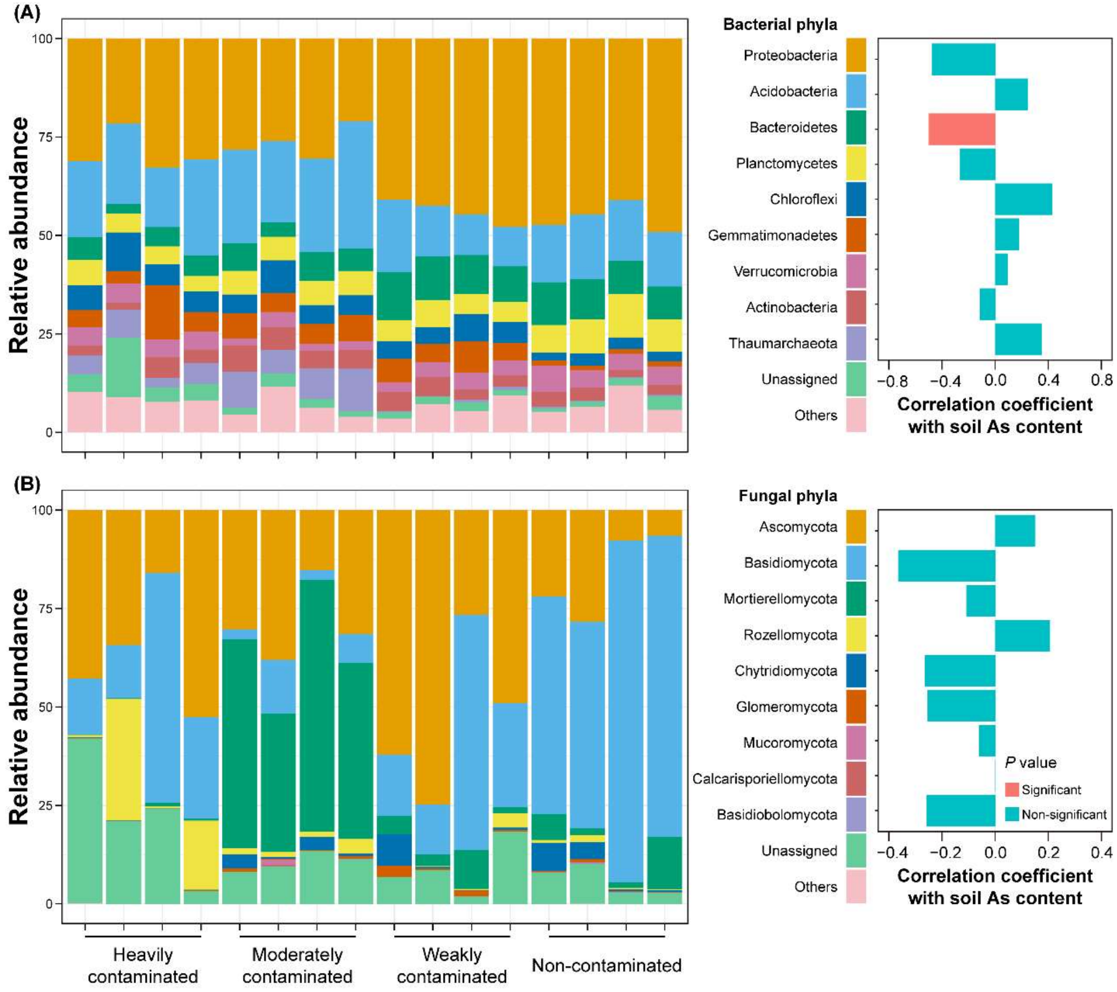

Supplementary Figure S4 | Relative abundances of bacterial and fungal phyla significantly correlated with arsenic content in *Pteris vittata* rhizosphere soil in the field. The bar plots on the right show the correlation coefficients between the relative abundance of these phyla and the soil arsenic content. The colors of the bars indicate the significance levels of the correlations between the relative abundance of these phyla and soil arsenic content.

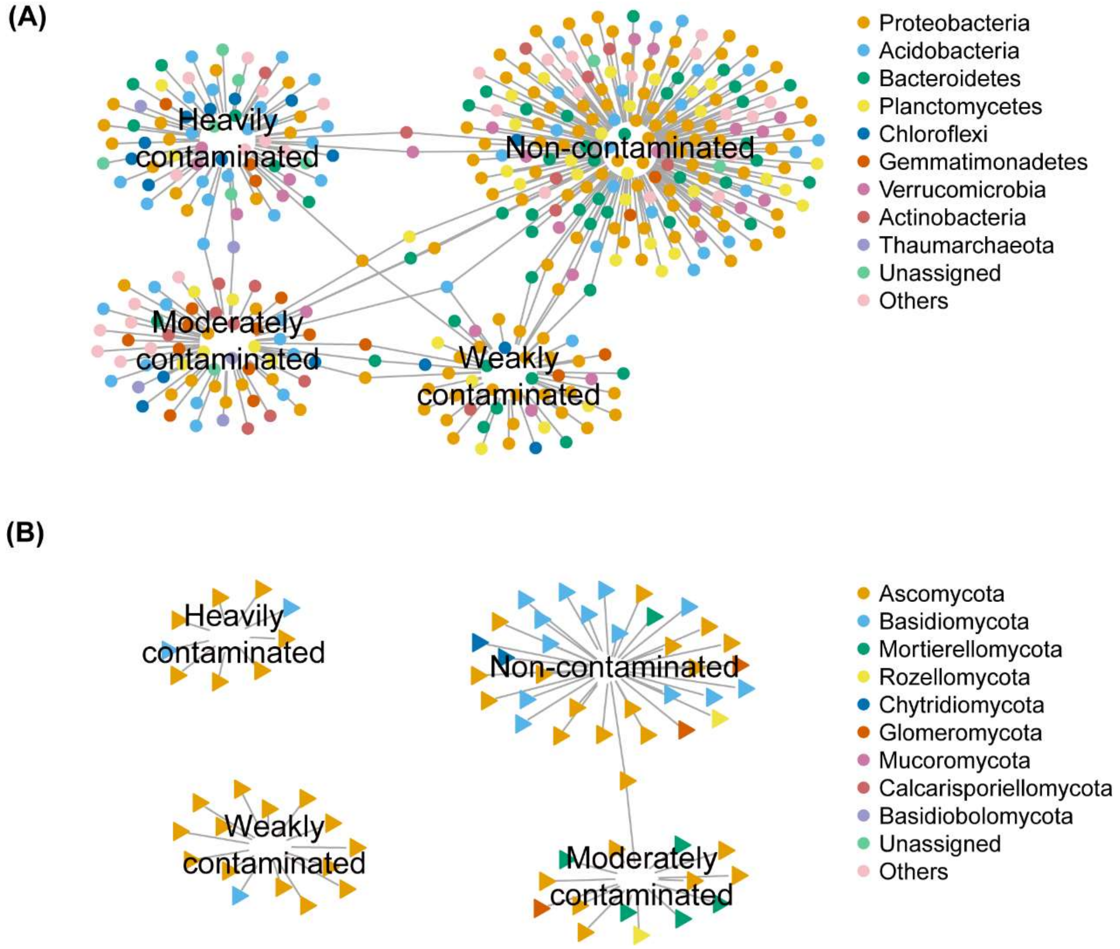

Supplementary Figure S5 | Bipartite networks display arsenic contamination-specific ASVs in soil bacterial and fungal communities determined using indicator species analysis. Circles represent individual bacteria, and triangles represent fungi ASVs that are positively and significantly associated with one or more of the arsenic contamination levels ($p < 0.05$). ASVs are colored according to their taxonomic assignment at the phylum level.

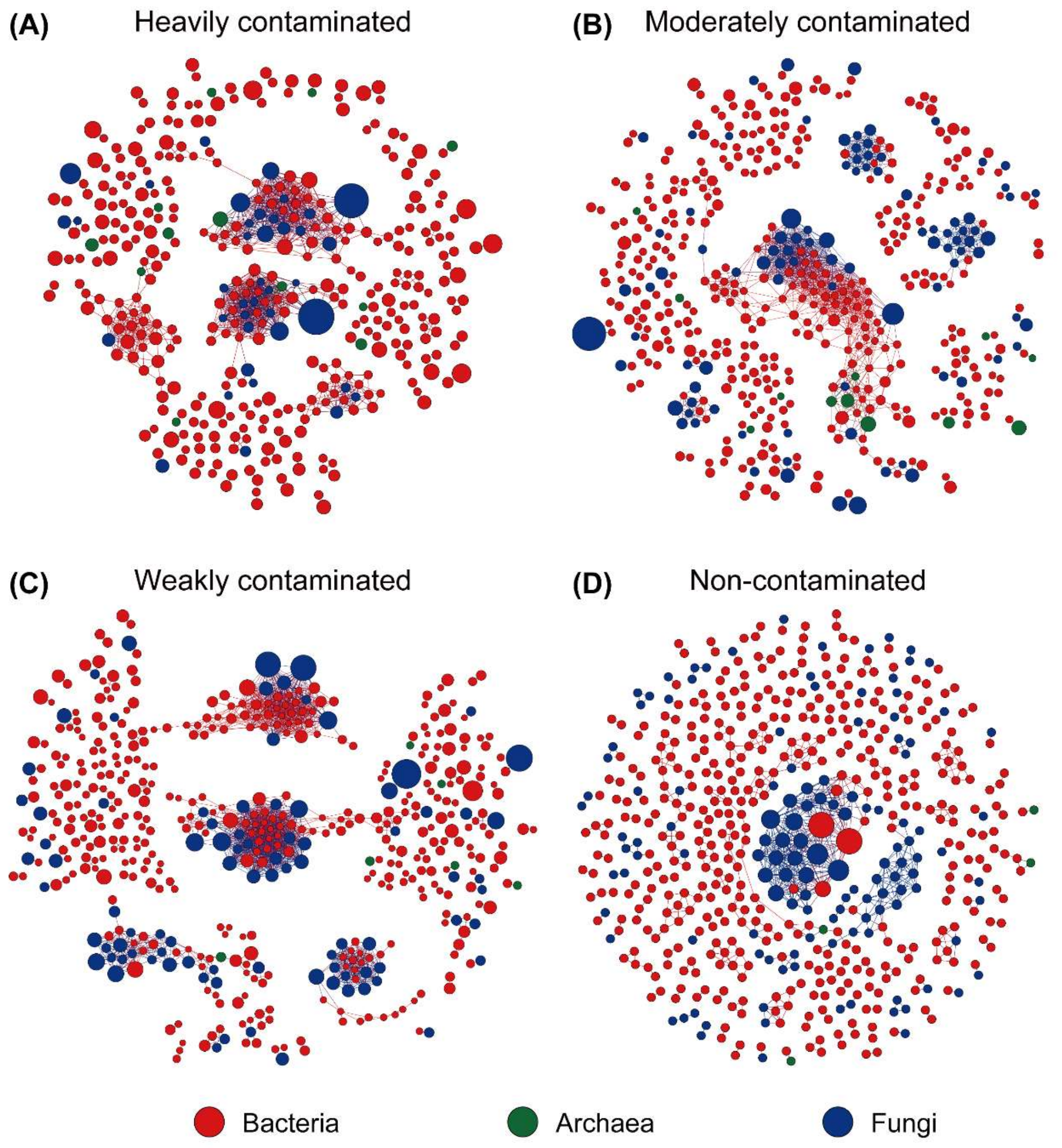

Contamination level	Modularity	No. of nodes	No. of links	No. of B-B links	No. of B-F links	No. of F-F links	Mean degree	Density	Centralization
Heavily-contaminated	0.75	392	1329	462	539	328	6.78	0.017	0.064
Moderately-contaminated	0.74	504	1474	788	449	237	5.85	0.012	0.064
Weakly-contaminated	0.75	508	2038	960	834	244	8.02	0.016	0.065
Non-contaminated	0.87	562	869	413	183	273	3.09	0.006	0.037

Supplementary Figure S6 | Co-occurrence network analysis showing biological interactions between rhizosphere soil microbes of *Pteris vittata*. Edges between nodes indicate strong (Spearman's correlation coefficient > |0.65|) and significant ($p < 0.001$) connections. The size of the node is proportional to the number of connections to the corresponding nodes.

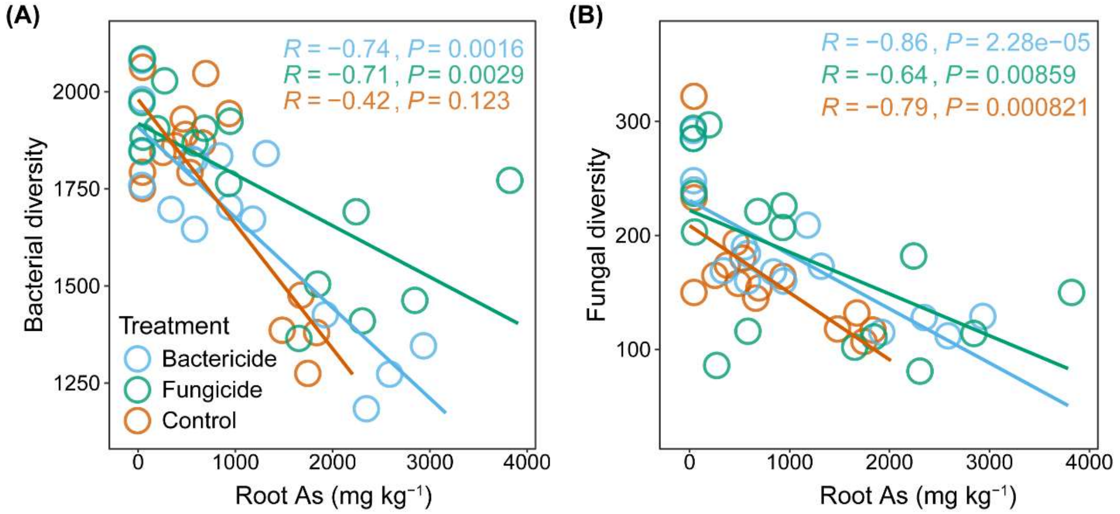

Supplementary Figure S7 | Relationship between root arsenic content and microbial diversity in pot experiments with bactericide and fungicide treatments.

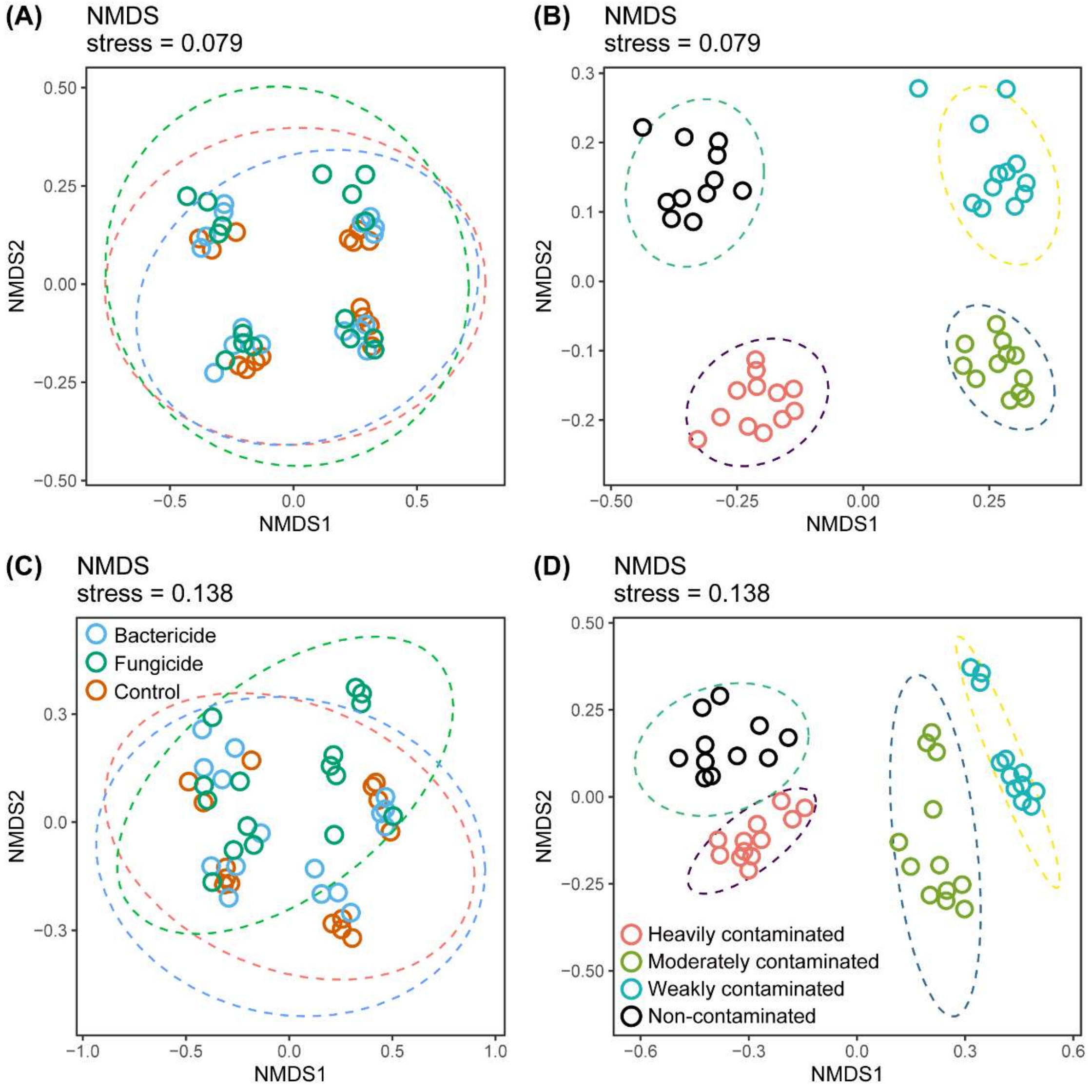

Supplementary Figure S8 | Nonmetric multidimensional scaling (NMDS) plot represents the difference of microbial community composition measured by Bray–Curtis distance with bactericide and fungicide treatments in pot experiments.

Supplementary Tables (S1−S7)

Supplementary Table S1. Arsenic concentration and partial physico-chemical properties of soil from the four sites.

Physico-chemical properties	Heavily-contaminated	Moderately-contaminated	Weakly-contaminated	Non-contaminated
As concentration($mg·kg^{-1}$)	24488±16148a	7617±2738b	1989±1893b	10.40±1.39b
pH	6.94±0.32ab	7.22±0.04a	6.95±0.25ab	6.57±0.12b
Electric conductivity (EC, S/m)	281±82.84a	249±52.75a	402±381a	201±44.91a
Total nitrogen (TN, $mg·kg^{-1}$)	322±122.66b	691±231b	698±289b	1351±510a
Nitrate-nitrogen (NO_3^--N, $mg·kg^{-1}$)	1.30±0.53b	1.69±0.62b	1.18±0.33b	6.48±1.49a
Ammonium-nitrogen (NH_4^+-N, $mg·kg^{-1}$)	5.38±1.24a	9.08±6.17a	26.82±27.27a	12.15±4.61a
Total phosphorus (TP, $mg·kg^{-1}$)	331±145b	2137±641a	852±579b	158±21.51b
Available phosphorus (AP, $mg·kg^{-1}$)	3.20±4.76a	16.78±7.04a	23.24±29.47a	9.14±6.03 a
Total potassium (TK, $mg·kg^{-1}$)	63026±52417a	11070±1309b	3950±2769b	43045±2289ab
Available potassium (AK, $mg·kg^{-1}$)	232±319a	265±42.35a	138±60.84a	257±100a

Note: mean and standard deviation from four replicates were shown; different letters indicate significant difference at $P<0.05$ level.

Supplementary Table S2. The abundance (normalized, %) of genes detected by AsChip and their corresponding functional processes.

Functional process	Gene	Heavily contaminated	Weakly contaminated	Non-contaminated
As(V) reduction	***arsC***	**0.0352**	**0.0067**	**0.0294**
	arsR	0.0020	0.0003	0.0023
Arsenic methylation and demethylation	***arsI***	**0.0005**	**0.0001**	**0.0003**
	arsM	0.0000	0.0000	0.0000
As(III) oxidation	*aoxA*	0.0003	0.0001	0.0005
	aoxB	0.0058	0.0010	0.0052
	aoxC	0.0000	0.0000	0.0000
	aoxR	**0.0004**	**0.0001**	**0.0003**
	aoxS	0.0010	0.0005	0.0012
Arsenic transport	*arsH*	0.0006	0.0001	0.0006
	acr3	0.0004	0.0005	0.0002
	arsA	0.0004	0.0001	0.0007
	arsB	0.0008	0.0002	0.0013
	arsD	0.0000	0.0000	0.0000
	arsP	**0.0001**	**0.0000**	**0.0000**

Note: Genes in bold were those shown in Figure 6.

Supplementary Table S3. Relative abundances of bacterial taxonomic groups in rhizosphere soil treated with bactericide/fungicide or untreated (control)

Treatments	Phylum/Genus	Control	Bactericide	Fungicide
Heavily-contaminated	**Proteobacteria**	28.46±1.47b	35.87±5.30a	35.37±3.38a
	Rhodoplanes	0.60±0.05b	0.80±0.16a	0.96±0.08a
	Bradyrhizobium	0.30±0.02b	0.66±0.16a	0.51±0.22ab
	Haliangium	1.20±0.21a	1.23±0.20a	0.64±0.12b
	Curvibacter	0.16±0.08b	0.11±0.04b	0.46±0.25a
	Pedomicrobium	0.13±0.02b	0.22±0.04a	0.16±0.02ab
	Devosia	0.22±0.06b	0.40±0.05a	0.19±0.07b
	Cupriavidus	0.19±0.03b	0.43±013a	0.28±0.07ab
	Methylobacillus	0.06±0.02b	0.07±0.02b	1.67±0.97a
	Thiobacillus	0.002±0.001ab	0.004±0.002a	0.0007±0.0007b
	Nordella	0.21±0.06b	0.37±0.03a	0.19±0.04b
	Hyphomicrobium	0.06±0.01b	0.12±0.04a	0.11±0.02a
	Reyranella	0.12±0.02a	0.13±0.03a	0.06±0.01b
	Hydrogenophaga	0.02±0.006b	0.03±0.01ab	0.05±0.02a
	Altererythrobacter	0.04±0.008b	0.07±0.02a	0.05±0.02ab
	Methyloversatilis	0.02v0.008b	0.02±0.004b	0.07±0.04a
	**Planctomycetes**	4.74±1.05a	3.43±0.26b	4.00±0.47ab
	Verrucomicrobia	5.92±0.47a	4.17±0.92b	5.34±0.22a
	Lacunisphaera	0.95±0.15a	0.35±0.17b	0.60±0.13b
	**Actinobacteria**	2.42±0.61b	6.33±1.26a	2.74±0.84b
	Pseudolabrys	0.25±0.03b	0.45±0.13a	0.36±0.10ab
	Gaiella	0.19±0.03b	0.36±0.07a	0.16±0.03b
	Streptomyces	0.20±0.06b	0.64±0.15a	0.24±0.13b
	Crossiella	0.18±0.10b	0.47±0.16a	0.16±0.05b
	Conexibacter	0.05±0.02b	0.21±0.09a	0.07±0.04b
	Iamia	0.01±0.003b	0.06±0.05a	0.02±0.01ab
	Solirubrobacter	0.03±0.002b	0.08±0.01a	0.03±0.01b
	**Patescibacteria**	1.55±0.63a	0.14±0.05b	0.65±0.17b
	**Nitrospirae**	0.49±0.03b	0.49±0.11b	0.82±0.10a
	Nitrospira	0.48±0.04b	0.47±0.09b	0.79±0.10a
	**Omnitrophicaeota**	0.17±0.03b	0.05±0.02b	0.35±0.12a
	**Elusimicrobia**	0.37±0.05b	0.23±0.10c	0.50±0.06a
	**Latescibacteria**	0.07±0.02a	0.04±0.01b	0.05±0.01ab
	**Dependentiae**	0.13±0.02b	0.07±0.02b	0.20±0.04a
	**Hydrogenedentes**	0.002±0.002b	0.005±0.002ab	0.02±0.01a
	**Bacteroidetes**	10.60±0.67a	8.04±2.51a	8.33±1.22a
	Flavisolibacter	0.21±0.08ab	0.29±0.03a	0.13±0.02b
Moderately-contaminated	**Bacteroidetes**	10.33±1.5a	6.83±1.22b	4.2±0.90c
	Ohtaekwangia	0.72±0.06a	0.51±0.20a	0.28±0.05b
	Terrimonas	1.36±0.23a	0.82±0.10b	0.53±0.14b

	**Verrucomicrobia**	4.50±1.42a	3.11±0.25a	3.44±0.54a
	Alterococcus	0.12±0.04b	0.12±0.02ab	0.18±0.02a
	**Planctomycetes**	9.55±0.91a	7.33±1.02b	7.10±0.91b
	Pirellula	1.08±0.12a	0.94±0.13ab	0.80±0.15b
	Firmicutes	0.17±0.04a	0.09±0.02b	0.14±0.05b
	Tumebacillus	0.03±0.01a	0.008±0.003b	0.020.006ab
	**Thaumarchaeota**	9.77±1.64b	15.34±2.11a	16.68±2.39a
	**Armatimonadetes**	0.96±0.18a	0.56±0.09b	0.57±0.05b
	**Euryarchaeota (Archaea)**	0.11±0.07a	0.13±0.03a	0.18±0.10a
	Methanothermobacter	0.005±0.004b	0.01±0.01ab	0.06±0.04a
	**Atribacteria**	0.0004±0.0008ab	0b	0.002±0.001a
	**Proteobacteria**	21.65±2.70a	21.90±0.40	23.45±4.68a
	Bdellovibrio	0.13±0.02ab	0.10±0.03b	0.18±0.05a
	Sphingomonas	0.67±0.08b	1.31±0.25a	0.97±0.25ab
	Haliangium	0.84±0.13a	0.75±0.08a	0.50±0.14b
	Methylobacillus	0.02±0.006b	0.03±0.007b	0.09±0.05a
	Bdellovibrio	0.13±0.02ab	0.10±0.003b	0.18±0.05a
	Polycyclovorans	0.30±0.05a	0.15±0.09b	0.11±0.05b
	Reyranella	0.09±0.03a	0.07±0.02ab	0.05±0.008b
	Phaselicystis	0.05±0.009a	0.03±0.006b	0.02±0.007b
	Pajaroellobacter	0.07±0.006a	0.06±0.01ab	0.05±0.02b
	Sandaracinus	0.05±0.01b	0.08±0.01a	0.07±0.008ab
	**Acidobacteria**	27.37±2.8a	29.49±1.86a	28.00±2.30a
	Gaiella	0.21±0.04a	0.15±0.02b	0.10±0.03b
	Stenotrophobacter	0.16±0.02a	0.11±0.01b	0.15±0.03ab
	Blastocatella	0.07±0.02a	0.03±0.002b	0.06±0.02ab
Weakly-contaminated	**Proteobacteria**	28.78±2.00b	30.36±2.01b	35.47±3.65a
	Sphingomonas	1.55±0.33	1.65±0.16a	0.65±0.20b
	Cupriavidus	0.09±0.02b	0.16±0.03a	0.13±0.05ab
	Geobacter	0.03±0.01a	0.02±0.01a	0.08±0.04b
	Thiobacillus	0.45±0.17ab	1.20±0.47a	0.59±0.33ab
	Polycyclovorans	0.22±0.11b	0.50±0.24a	0.05±0.01b
	Pseudoduganella	0.03±0.01b	0.03±0.01b	0.08±0.04a
	Aquicella	0.30±0.10a	0.29±0.04a	0.15±0.06b
	Nordella	0.07±0.01b	0.13±0.03a	0.05±0.02b
	Hydrogenophaga	0.05±0.01b	0.72±0.51a	0.07±0.02b
	**Acidobacteria**	22.90±1.14a	19.86±2.38a	15.77±2.30b
	**Bacteroidetes**	13.52±2.58a	14.11±0.68a	9.56±1.24a
	Ohtaekwangia	2.05±0.63a	2.46±0.18a	0.69±0.37b
	Terrimonas	1.16±0.27a	1.23±0.24a	0.44±0.14b
	Niastella	0.50±0.16a	0.47±0.06a	0.20±0.06b
	Flavisolibacter	0.12±0.03a	0.14±0.03a	0.04±0.02b
	**Planctomycetes**	9.32±1.00a	8.52±0.86a	9.31±0.48a
	Pirellula	1.16±0.22a	1.24±0.08a	0.85±0.08b

	**Verrucomicrobia**	5.50±0.68a	6.58±1.23a	6.06±1.55a
	Opitutus	0.27±0.06ab	0.21±0.05b	0.38±0.11ab
	Chthoniobacter	0.21±0.01a	0.11±0.03b	0.19±0.07ab
	Alterococcus	0.16±0.07b	0.08±0.03b	0.45±0.18a
	**Actinobacteria**	4.00±0.90a	3.69±0.79a	2.72±1.67a
	Gaiella	0.54±0.08a	0.63±0.14a	0.23±0.06b
	**Gemmatimonadetes**	2.48±0.31a	3.03±0.15a	3.09±0.84a
	Bdellovibrio	0.30±0.10ab	0.19±0.03b	0.44±0.18a
	**Armatimonadetes**	0.47±0.03a	0.36±0.09b	0.34±0.04b
	**Nitrospirae**	0.36±0.09b	0.64±0.11a	0.38±0.21ab
	Nitrospira	0.31±0.07b	0.59±0.10a	0.36±0.20ab
	**Omnitrophicaeota**	0.28±0.13b	0.33±0.16b	1.02±0.52a
	**Hydrogenedentes**	0.04±0.008b	0.05±0.01b	0.11±0.04a
	**Fibrobacteres**	0.02±0.02b	0.005±0.004b	0.07±0.04a
	**Spirochaetes**	0.005±0.001b	0.002±0.001b	0.03±0.02a
	**Kiritimatiellaeota**	0.0005±0.0008b	0.001±0.001b	0.01±0.01a
Non-contaminated	**Proteobacteria**	49.16±3.70a	48.25±5.97a	46.35±4.96a
	Dongia	1.30±0.07a	0.87±0.14b	0.66±0.08b
	Pedomicrobium	0.28±0.007ab	0.24±0.03b	0.40±0.12a
	Allorhizobium-Neorhizobium-Pararhizobium-Rhizobium	0.45±0.10a	0.30±0.09ab	0.25±0.02b
	Hyphomicrobium	0.16±0.03ab	0.13±0.01b	0.22±0.07a
	Lysobacter	0.52±0.06a	0.35±0.07b	0.30±0.08b
	Reyranella	0.17±0.02ab	0.21±0.04a	0.14±0.02b
	Hirschia	0.16±0.06a	0.18±0.01a	0.06±0.03b
	Noviherbaspirillum	0.09±0.04b	0.22±0.09ab	0.25±0.05a
	Mesorhizobium	0.16±0.04a	0.07±0.02b	0.08±0.02b
	**Acidobacteria**	16.25±1.43a	17.68±4.39a	20.11±2.96a
	Sphingomonas	8.20±2.31a	5.97±2.95ab	3.51±0.90b
	**Bacteroidetes**	5.53±1.09a	5.56±0.71a	3.15±0.35b
	Ohtaekwangia	0.24±0.07a	0.14±0.02b	0.08±0.02b
	Flavitalea	0.11±0.02a	0.07±0.02ab	0.05±0.005b
	**Actinobacteria**	4.78±0.70a	3.96±1.34a	4.00±0.52a
	Gaiella	0.51±0.07a	0.35±0.03b	0.35±0.04b
	Conexibacter	0.07±0.02b	0.13±0.03a	0.08±0.02ab
	Iamia	0.07±0.02a	0.03±0.03ab	0.02±0.01b
	**Gemmatimonadetes**	3.56±0.32a	2.99±0.56a	1.63±0.41b
	Gemmatimonas	1.30±0.07a	1.22±0.50a	0.57±0.17b
	**Patescibacteria**	0.14±0.04b	0.16±0.04b	0.26±0.07a
	**Chlamydiae**	0.10±0.01b	0.11±0.04b	0.18±0.03a
	**Elusimicrobia**	0.08±0.02b	0.09±0.04ab	0.16±0.04a

Note: mean and standard deviation from four replicates were shown; different letters indicate significant difference at $P<0.05$ level.

Supplementary Table S4. Relative abundances of FAPROTAX predicted ecological functions in rhizosphere soil treated with bactericide/fungicide or untreated (control)

Treatments	Functions	Control	Bactericide	Fungicide
Heavily-contaminated	AC	0.18±0.03ab	0.19±0.03a	0.13±0.01b
	PoE	0.02±0.003a	0.02±0.003b	0.01±0.002c
	ANO	0.007± 0.0005a	0.005±0.001b	0.008±0.001a
	ME	0.001± 0.0002b	0.001±0.0003b	0.02±0.001a
	MO	0.001± 0.0002b	0.001±0.0002b	0.002±0.01a
	RoSC	0.005±0.002a	0.003±0.001ab	0.002±0.000b
	SR	0.0006±0.0001a	0.0004±0.0001b	0.0005±0.0001ab
	CH	0.0006±0.0001a	0.003±0.001ab	0.002±0.0001b
	APoS	0.0006±0.0001a	0.0005± 9.19E-05ab	0.0004± 3E-05
	RA	7.91E-05± 2.7E-05a	4.12E-05± 1.97E-05b	0.00002± 6.08E-06b
	RC	5.66E-05± 2.11E-05a	4.56E-05± 9.32E-06ab	2.2E-05± 1.78E-05b
	CR	0b	1.98E-05± 4.8E-06ab	2.42E-05± 1.75E-05ab
	AnMHD	0b	3.25E-06± 5.63E-06ab	7.88E-06± 4.69E-06a
Moderately-contaminated	CHe	0.11±0.01a	0.09±0.01b	0.08±0.01b
	AC	0.09±0.01a	0.07±0.001b	0.06±0.01b
	Nitrate R	0.02±0.004	0.01±0.002b	0.02±0.003b
	PoE	0.02±0005a	0.02±0.002b	0.01±0.002b
	Nitrate R	0.01±0.002a	0.01±0.001b	0.01±0.001b
	Nitrogen R	0.01±0.002a	0.01±0.001b	0.01±0.001b
	PH	0.01±0.003a	0.01±0.002b	0.01±0.001ab
	Nitrate D	0.01±0.002a	0.005±0.001b	0.005±0.001b
	Nitrite D	0.01±0.002a	0.005±0.001b	0.005±0.001b
	NoD	0.01±0.002a	0.005±0.001b	0.005±0.001b
	DE	0.01±0.002a	0.005±0.001b	0.005±0.001b
	Nitrite R	0.01±0.002a	0.005±0.001b	0.005±0.001b
	APSo	0.01±0.002a	0.005±0.001b	0.005±0.001b
	AP	0.01±0.002a	0.005±0.001b	0.005±0.001b
	ME	0.001±0.0001b	0.001±0.0002b	0.002±0.001a
	MO	0.001±0.0001b	0.001±0.0002b	0.002±0.001a
	DHO	0.001±0.0002b	0.001±0.0001b	0.002±0.0009a
	RoSC	0.006±0.0003a	0.004±0.0008b	0.003±0.0006c
	SR	0.005±0.0005a	0.004±0.0008b	0.003±0.0005c
	MAO	0.003±0.001a	0.001±0.0002b	0.001±0.0005b
	HM	0.0002±0.0001b	0.0003±0.0002ab	0.001±0.001a
	MET	0.0002±0.0001b	0.0003±0.0002ab	0.001±0.001a
	MB CO_2 RW H_2	0.0002±0.0001b	0.0003±0.0002ab	0.001±0.0001a

	APoS	0.001±0.0003a	0.0002±0.0001b	0.0002± 2.64E-05b
	CE	0.001± 8.93E-05ab	0.001±0.0003a	0.0006±0.0002b
	ACD	0.001±0.0002a	0.0002± 6.06E-05b	0.0001± 7.12E-05b
	HD	0.001±0.0001a	0.0002± 7.92E-05b	0.0003± 5.32E-05b
	HPA	0.0005±0.0002a	0.0001± 8.74E-05b	0.0001± 2.23E-05b
	HA	0.0005±0.0002a	0.0001± 8.74E-05b	0.0001± 2.23E-05b
Weakly-contaminated	CHe	0.16±0.04ab	0.21±0.02a	0.16±0.02b
	AC	0.15±0.04ab	0.19±0.02a	0.12±0.02b
	FE	0.01±0.002b	0.01±0.003b	0.02±0.005a
	ANO	0.01±0.003b	0.01±0.002a	0.01±0.004b
	ME	0.002±0.0004b	0.002±0.0004b	0.01±0.01a
	DOoSC	0.01±0.01b	0.03±0.01a	0.01±0.01b
	MO	0.002±0.001b	0.002±0.0005b	0.01±0.01a
	DSO	0.0004±0.0002b	0.001±0.0002a	0.01±0.01b
	HPA	0.0004±0.0002b	0.001±0.0002a	0.0003±0.0002b
	HA	0.0004±0.0002b	0.001±0.0002a	0.0003±0.0002b
Non-contaminated	CHe	0.22±0.01a	0.21±0.03ab	0.17±0.02b
	AC	0.21±0.01a	0.19±0.03ab	0.16±0.01b
	PH	0.03±0.002b	0.03±0.002b	0.03±0.003a
	PHo	0.03±0.002b	0.03±0.002b	0.03±0.003a
	CY	0.004±0.0007b	0.005±0.001b	0.01±0.003a
	OP	0.004±0.0007b	0.005±0.001b	0.01±0.003a
	DSO	7.58E05± 2.66E-05ab	0.0001± 3.87E-05a	4.53E-05± 2.12E-05b
	CH	0.006±0.0002a	0.004±0.0005ab	0.003±0.001b

Note: mean and standard deviation from four replicates were shown; different letters indicate significant difference at $P<0.05$ level. "**Functions**" were abbreviation of details on **Table S5**.

Supplementary Table S5. Rhizosphere bacteria with predicted ecological functions

Abbreviation	Details
AC	Aerobic_chemoheterotrophy
PoE	Predatory_or_exoparasitic
ANO	Aerobic_nitrite_oxidation
ME	Methylotrophy
MO	Methanol_oxidation
RoSC	Respiration_of_sulfur_compounds
SR	Sulfate_respiration
CH	Chitinolysis
APoS	Animal_parasites_or_symbionts
RA	Reductive_acetogenesis
RC	Sulfite_respiration
CR	Chlorate_reducers
AnMHD	Aliphatic_non_methane_ Hydrocarbon_degradation
CHe	Chemoheterotrophy
Nitrate R	Nitrate_reduction
Nitrate R	Nitrate_respiration
Nitrogen R	Nitrogen_respiration
PH	Photoheterotrophy
Nitrate D	Nitrate_denitrification
Nitrite D	Nitrite_denitrification
NoD	Nitrous_oxide_denitrification
DE	Denitrification
Nitrite R	Nitrite_respiration
APSo	Anoxygenic_photoautotrophy_ S_oxidizing
AP	Anoxygenic_photoautotrophy
DHO	Dark_hydrogen_oxidation
MAO	Manganese_oxidation
HM	Hydrogenotrophic_methanogenesis
ME	Methanogenesis
MB CO_2 RW H_2	Methanogenesis_by_CO_2 _reduction_with_H_2
APoS	Animal_parasites_or_symbionts
CE	Cellulolysis
ACD	Aromatic_compound_degradation
HD	Hydrocarbon_degradation
HPA	Human_pathogens_all
HA	Human_associated
FE	Fermentation
DOoSC	Dark_oxidation_of_sulfur_compounds
DSO	Dark_sulfide_oxidation
PH	Phototrophy
PHo	Photoautotrophy
CY	Cyanobacteria
OP	Oxygenic_photoautotrophy
CH	Chitinolysis

Supplementary Table S6. Relative abundances of fungal taxonomic groups in rhizosphere soil treated with bactericide/fungicide or untreated (control)

Treatments	Phylum/Genus	Control	Bactericide	Fungicide
Heavily-contaminated	**Ascomycota**	69.60±4.24a	80.11±6.73a	73.80±8.18a
	Penicillium	8.56±1.88a	10.27±2.63a	3.57±2.55b
	Myrothecium	0.02±0.02b	0.19±0.14a	0.003±0.002b
	**Mortierellomycota**	1.02±0.42a	0.38±0.28b	0.15±0.09b
	Mortierella	1.02±0.38a	0.38±0.25b	0.14±0.08b
Moderately-contaminated	**Ascomycota**	48.99±20.04a	63.05±7.21a	64.99±17.06a
	Trichoderma	0.01±0.01a	0.03±0.01a	0.05±0.04a
	Pyrenochaeta	2.11±1.82ab	3.35±1.90a	0.26±0.34b
	**Glomeromycota**	24.36±11.11a	3.46±3.25b	7.84±7.24b
	**Rozellomycota**	0.52±0.47b	0.76±0.51b	3.77±1.69a
	**Mortierellomycota**	4.30±1.52b	14.53±8.49a	2.62±2.46b
	Mortierella	4.30±1.52b	14.53±8.49a	2.62±2.46b
Weakly-contaminated	**Ascomycota**	62.46±9.07a	61.51±7.21a	65.48±22.79a
	Penicillium	0.02±0.01ab	0.03±0.01a	0.01±0.01b
	**Basidiomycota**	10.96±6.57a	6.28±4.05ab	1.08±1.28b
Non-contaminated	**Rozellomycota**	8.15±8.09ab	4.15±3.01b	20.46±11.23a
	**Chytridiomycota**	0.07±0.03b	0.15±0.03ab	0.23±0.10a

Note: mean and standard deviation from four replicates were shown; different letters indicate significant difference at $P<0.05$ level.

Supplementary Table S7. Relative abundances of fungal functional guilds in rhizosphere soil treated with bactericide/fungicide or untreated (control)

Treatments	Functions	Control	Bactericide	Fungicide
Moderately-contaminated	Arbuscular Mycorrhizal	24.36±11.11a	3.46±3.25b	7.83±7.24b
Weakly-contaminated	Animal Pathogen	0.21±0.06ab	0.36±0.21a	0.08±0.09b
	Soil Saprotroph	0.07±0.06b	0.30±0.10a	0.05±0.08b
Non-contaminated	Animal Pathogen	0.10±0.04a	0.03±0.02b	0.08±0.03ab
	Soil Saprotroph	0.10±0.04ab	0.03±0.02b	0.14±0.08a
	Plant Saprotroph	0.01±0.01ab	0.02±0.01a	0.003±0.003b

Note: mean and standard deviation from four replicates were shown; different letters indicate significant difference at *P*<0.05 level.
